# Melatonin Induces Analgesic Effects through MT_2_ Receptor-Mediated Neuroimmune Modulation in the Mice Anterior Cingulate Cortex

**DOI:** 10.34133/research.0493

**Published:** 2024-10-08

**Authors:** Jian Wang, Junxiang Gu, Fujuan Ma, Yi Wei, Pan Wang, Shanming Yang, Xianxia Yan, Yifan Xiao, Keke Xing, Anxin Lou, Liru Zheng, Tingting Cao, Dayu Zhu, Jinlian Li, Luoying Zhang, Yunqing Li, Tao Chen

**Affiliations:** ^1^ Department of Anatomy and K.K. Leung Brain Research Centre, Fourth Military Medical University, Xi’an 710032, China.; ^2^ Department of Neurosurgery, Tangdu Hospital, Fourth Military Medical University, Xi’an 710038, China.; ^3^ Department of Neurosurgery, the Second Affiliated Hospital of Xi’an Jiaotong University, Xi’an 710004, China.; ^4^ School of Medicine, Northwest University, Xi’an 710069, China.; ^5^ Key Laboratory of Molecular Biophysics of Ministry of Education, College of Life Science and Technology, Huazhong University of Science and Technology, Wuhan 430074, China.

## Abstract

Neuropathic pain (NP) represents a considerable clinical challenge, profoundly impacting patients’ quality of life. Presently, pharmacotherapy serves as a primary approach for NP alleviation, yet its efficacy often remains suboptimal. Melatonin (MLT), a biologically active compound secreted by the pineal gland, has long been associated with promoting and maintaining sleep. Although recent studies suggest analgesic effects of MLT, the underlying mechanism remains largely unknown, particularly its impact on the cortex. In this study, we induced an NP model in mice through spared nerve injury (SNI) and observed a considerable, dose-dependent alleviation in NP symptoms following intraperitoneal or anterior cingulate cortex (ACC) administration of MLT. Our findings further indicated that the NP management of MLT is selectively mediated by MLT-related receptor 2 (MT_2_R), rather than MT_1_R, on neurons and microglia within the ACC. Transcriptome sequencing, complemented by bioinformatics analysis, implicated MLT in the modulation of Gα(i) and immune-inflammatory signals. Specifically, MLT inhibited the excitability level of pyramidal cells in the ACC by activating the Gα(i) signaling pathway. Simultaneously, MLT attenuated M_1_ polarization and promoted M_2_ polarization of microglia, thereby mitigating the inflammatory response and type II interferon response within the ACC. These findings unveil a hitherto unrecognized molecular mechanism: an MLT-mediated neuroimmune modulation pathway in the ACC mediated by MT_2_R. This elucidation sheds light on the regulatory character of MLT in chronic nociceptive pain conditions, offering a prospective therapeutic strategy for NP management.

## Introduction

Neuropathic pain (NP) is a major contributor to severe and enduring chronic pain. Given the intricate mechanisms underlying its development, while existing pharmaceutical treatments, including antidepressants (amitriptyline, duloxetine), antiepileptic drugs (pregabalin), and opioids (morphine, etc.), do offer relief of pain in certain instances, their limitations and side effects highlight the need for more targeted and effective therapeutic options [1–3]. Extensive research has demonstrated a close correlation between NP processes and abnormal activity within the sensory modulation regions of the central nervous system [4,5]. Among these areas, the anterior cingulate cortex (ACC) plays a well-studied role in pain modulation [6–8]. Our previous studies align with this finding, revealing that NP triggers abnormal activation of neurons, particularly pyramidal neurons, within the ACC [9–11]. These activated pyramidal cells amplify the discharge activity of spinal dorsal horn neurons, contributing to enhanced pain sensitization [12]. Remarkably, suppressing the activation of pyramidal cells in the ACC yields substantial analgesic effects [13,14]. Therefore, the cortical regions of the ACC emerge as a promising therapeutic target for NP treatment.

Melatonin (MLT), a hormone released from the pineal body, emerges as a crucial regulator in promoting and maintaining sleep [15,16]. More recently, evidence has emerged indicating that MLT’s proficiency as an effective free radical scavenger, safeguarding neurons against the aggregation of reactive oxygen species (ROS) and alleviating neurotoxicity [17–19], thereby regulating a range of neurological diseases, encompassing Parkinson’s disease, Alzheimer’s disease, and various other conditions [20–22]. In addition, prior research has demonstrated that oral, intraperitoneal, or intrathecal administration of MLT, through the MT_2_ receptor (MT_2_R), exerts analgesic effects in rats with NP or chemotherapy pain in behavioral experiments [23–25]. MLT inhibits the protein expression of mitogen-activated protein kinases (MAPKs) in astrocyte and microglia in the spinal cord [25–27]. In our previous work, we also show that intraperitoneal injection of MLT alleviates NP through activation of MT_2_R and NOS1 in the dorsal root ganglia in mice [26]. However, the mechanism of MLT’s analgesic action remains largely unexplored, and there is a marked lack of studies examining its analgesic effect and mechanism in the cortex, especially in the ACC.

In the current study, we constructed an NP model of mice via spared nerve injury (SNI) and observed that administration of MLT, via either intraperitoneal injection or direct microinjection into the ACC, resulted in a notable, dose-dependent alleviation of NP. The mechanism underlying MLT’s effect involved the activation of MT_2_R expressed in both ACC pyramidal cells and microglia. This activation was characterized by a Gα(i)-mediated suppression of pyramidal cell activity, inhibition of microglial M_1_ polarization, and a reduction in the release of inflammatory cytokines within the ACC. These findings not only enhance our understanding of MLT’s analgesic mechanisms but also hold potential implications for clinical relief of NP.

## Results

### MLT induces analgesic effect through MT_2_R with a manner dependent on dosage in the ACC

**Fig. 1.**
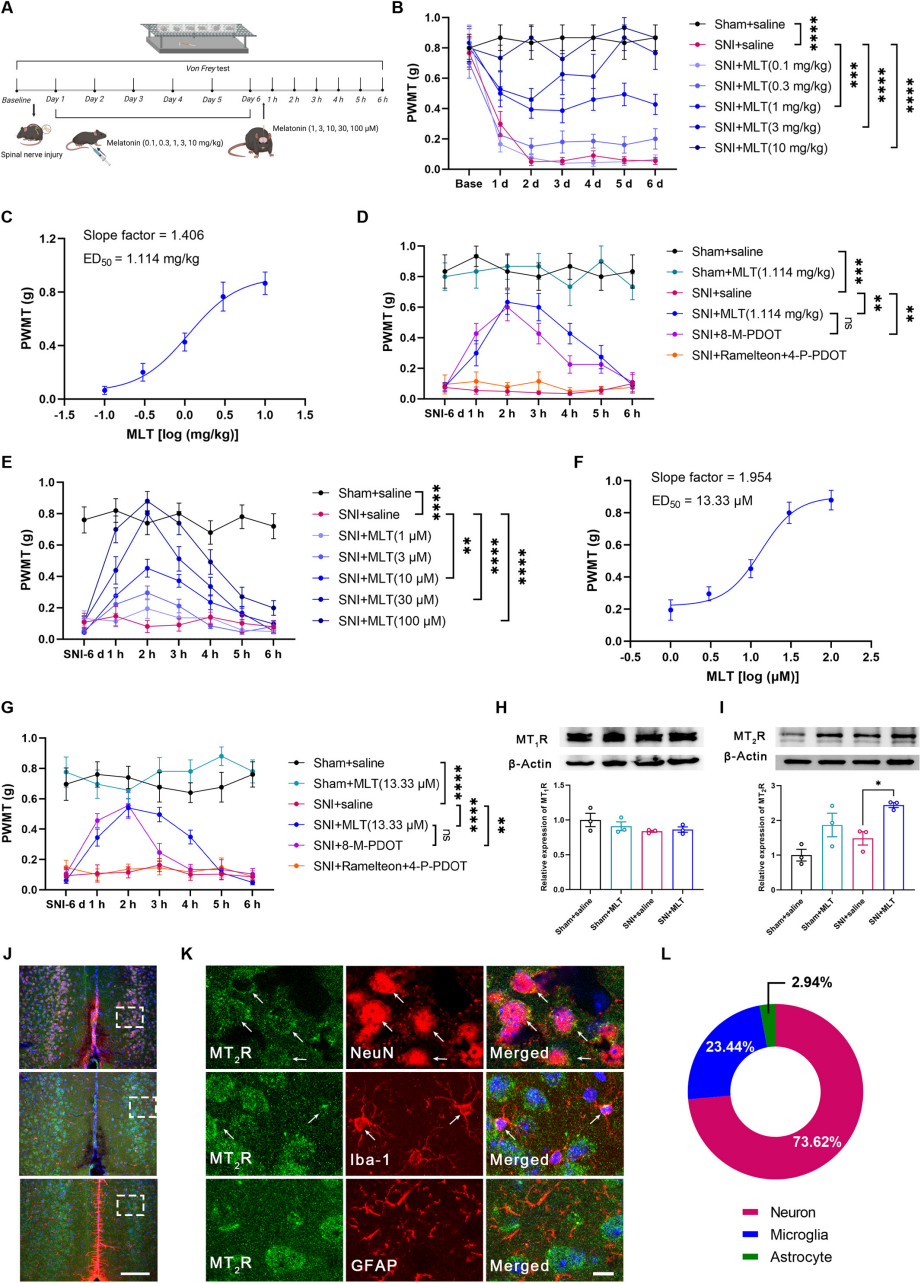
Administration of MLT induces analgesic effect through MT_2_R in SNI mice. (A) Experimental timeline. (B) The PWMT is tested for evaluation of the potential analgesic effect with intraperitoneal administration of MLT or saline (*n* = 6). (C) Dose–effect curves of MLT’s analgesic effects with intraperitoneal administration. (D) The PWMT is tested for evaluation of the potential analgesic effect with intraperitoneal administration of MLT (1.114 mg/kg), 8-M-PDOT (MT_2_R agonist, 1.3 mg/kg), Ramelteon (MT_1_R/MT_2_R agonist, 1.4 mg/kg) + 4-P-PDOT (MT_2_R antagonist, 0.14 mg/kg), or saline (*n* = 6). (E) The PWMT is tested for evaluation of the potential analgesic effect with ACC microinjection of MLT or saline (*n* = 10). (F) Dose–effect curves of MLT’s analgesic effects with ACC microinjection. (G) The PWMT is tested for evaluation of the potential analgesic effect with ACC microinjection of MLT (13.33 μM), 8-M-PDOT (10 μM), Ramelteon (10 μM) + 4-P-PDOT (10 μM), or saline (*n* = 10). (H and I) WB showing the expression of MT_1_R protein and MT_2_R in the ACC of each group mice (*n* = 3). (J and K) Immunofluorescence staining for MT_2_R/NeuN/Iba-1/GFAP in the ACC. Scale bars, 200 μm (J) and 10 μm (K). (L) Ratio of MT_2_R-immunoreactivities in NeuN-, Iba-1-, and GFAP-immunoreactive cells (*n* = 3). *P < 0.05; **P < 0.01; ***P < 0.001; ****P < 0.0001. Figure 1A was created with BioRender.com, with permission.

We used the SNI model to induce NP in adult male mice, and the paw withdrawal mechanical thresholds (PWMTs) were then evaluated in von Frey filament test, as stated in our earlier studies (Fig. 1A) [28,29]. We observed a significant decrease in PWMTs from day 1 to day 6 after surgery in SNI mice compared to sham mice (Fig. 1B). To investigate the possible analgesic effects of MLT, we administered varying concentrations of MLT intraperitoneally (0.1, 0.3, 1, 3, or 10 mg/kg) or via microinjection (1 μM, 3 μM, 10 μM, 30 μM, 100 μM, 0.5 μl) into the ACC (Fig. 1B and E). The results demonstrated a pain-relieving effect of MLT that varied according to the dose administered, with an ED_50_ value (the median effective dose necessary to elicit an analgesic response) of 1.114 mg/kg for intraperitoneal administration and 13.33 μM for microinjection into the ACC (Fig. 1C and F). We also tested the impact of MLT at ED_50_ concentration on pain sensation in female mice and found that MLT, either by intraperitoneal injection or by ACC microinjection, alleviated the mechanical allodynia of SNI female mice (Fig. S1). Accordingly, we believe that MLT’s analgesia effect is applicable to both male and female mice.

To test whether MLT’s effect is mediated by MT_1_R or MT_2_R, we administered, through intraperitoneal or by ACC microinjection, MT_2_R agonist 8-M-PDOT [the pKi values (affinity constant between MLT and its target) binding to MT_1_R and MT_2_R were 8.23 and 8.95, respectively] and found that 8-M-PDOT mimicked MLT’s analgesic effect. However, when MT_1_R was selectively activated, by using a combined approach of Ramelteon (MT_1_R and MT_2_R agonists) and 4-P-PDOT (MT_2_R antagonist), no significant alterations in PWMTs were detected (Fig. 1D and G). These results hint at that MLT exerts a notable analgesic effect in the ACC, mediated primarily by MT_2_R rather than by MT_1_R.

Subsequently, the expression intensity of MT_1_R and MT_2_R in the ACC was tested through Western blot (WB) analysis. It was observed that the MT_1_R expression remained unchanged in both SNI mice and SNI mice receiving intraperitoneal administration of MLT (Fig. 1H). However, MT_2_R expression was significantly up-regulated in SNI mice and this up-regulation was reversed by MLT administration (Fig. 1I), further supporting the involvement of ACC MT_2_R in MLT’s analgesia. Interestingly, immunofluorescent staining revealed that the MT_2_R immunoreactivity was mainly localized to neurons (73.62%) and microglia (23.44%) (Fig. 1J to L), strongly suggesting that MLT may exert its effect through both neuronal and microglial MT_2_R in the ACC.

### MLT regulates the immune-inflammatory response through G protein-coupled receptor signaling and type II interferon signaling

**Fig. 2.**
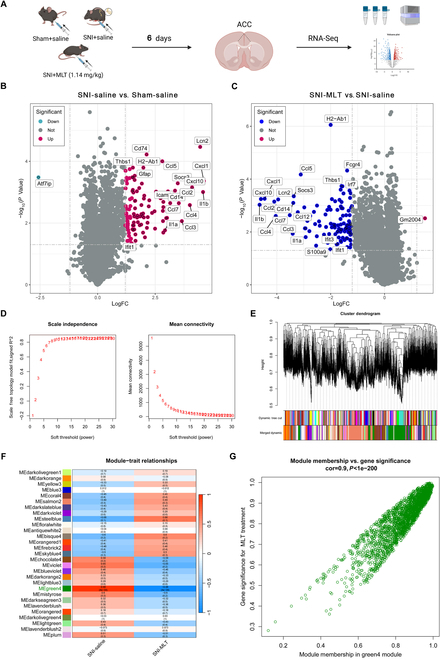
Phenotypic-related genes obtained via differential gene analysis and WGCNA. (A) Schematic illustrating the RNA-seq procedure. (B and C) Volcano plot depicting changes in transcript expression in the ACC, within SNI + saline versus sham + saline mice (B), and SNI + MLT versus SNI + saline mice (C) (*n* = 3). The *y* axis corresponds to −log_10_ (*P* value), and the *x* axis displays log_2_ fold change (logFC). Red dots represent up-regulated transcripts (*P* < 0.05, logFC > 1.2), and blue dots represent down-regulated transcripts (*P* < 0.05, logFC < −1.2). The dashed line parallel to the *x* axis indicates a raw *P* value = 0.05. The dashed line parallel to the *y* axis indicates a raw FC = 1.2. (D) Scale-free index analysis for soft-threshold power and mean connectivity analysis for various soft-threshold powers. (E) Module clustering dendrogram based on a dissimilarity measure (1-TOM). (F) Heatmap displaying the correlation between module eigengenes and MLT treatment. (G) Scatterplot of GS for MLT treatment versus MM in the green4 module. Figure 2A was created with BioRender.com, with permission.

The molecular principles behind the analgesic effect of MLT remain unknown. To elucidate this mechanism, we studied primary regulatory messages intensively based on transcriptome sequencing technology [30]. Homogenates of the ACC were arranged from sham-saline, SNI-saline, and SNI-MLT mice, and the precipitated RNA was subjected to sequencing (Fig. 2A). After normalization (Fig. S2A and B), the analysis of RNA sequencing data was conducted to pinpoint genes that exhibited a significant (*P* < 0.05) enrichment of approximately 1.2-fold or more in the precipitated RNA from ACC homogenates across sham-saline, SNI-saline, and SNI-MLT groups. The results revealed 107 differentially expressed genes (DEGs; 106 up-regulated and 1 down-regulated) totally in the SNI-saline versus sham-saline group (Fig. 2B), and in total 109 DEGs (1 up-regulated and 108 down-regulated) in the SNI-MLT versus SNI-saline group (Fig. 2C). Subsequently, employing weighted gene co-expression network analysis (WGCNA) for module classification, we identified 29 modules (Fig. 2D and E) after cluster analysis (Fig. S2C). By assessing the association coefficient between modules and MLT intervention, the green4 module, exhibiting the highest correlation (*r* = 0.98, *P* = 4 × 10^−4^) with MLT intervention, was chosen as the key module (Fig. 2F). From the green4 module, we identified 2,223 hub genes for subsequent analysis (Fig. 2G).

**Fig. 3.**
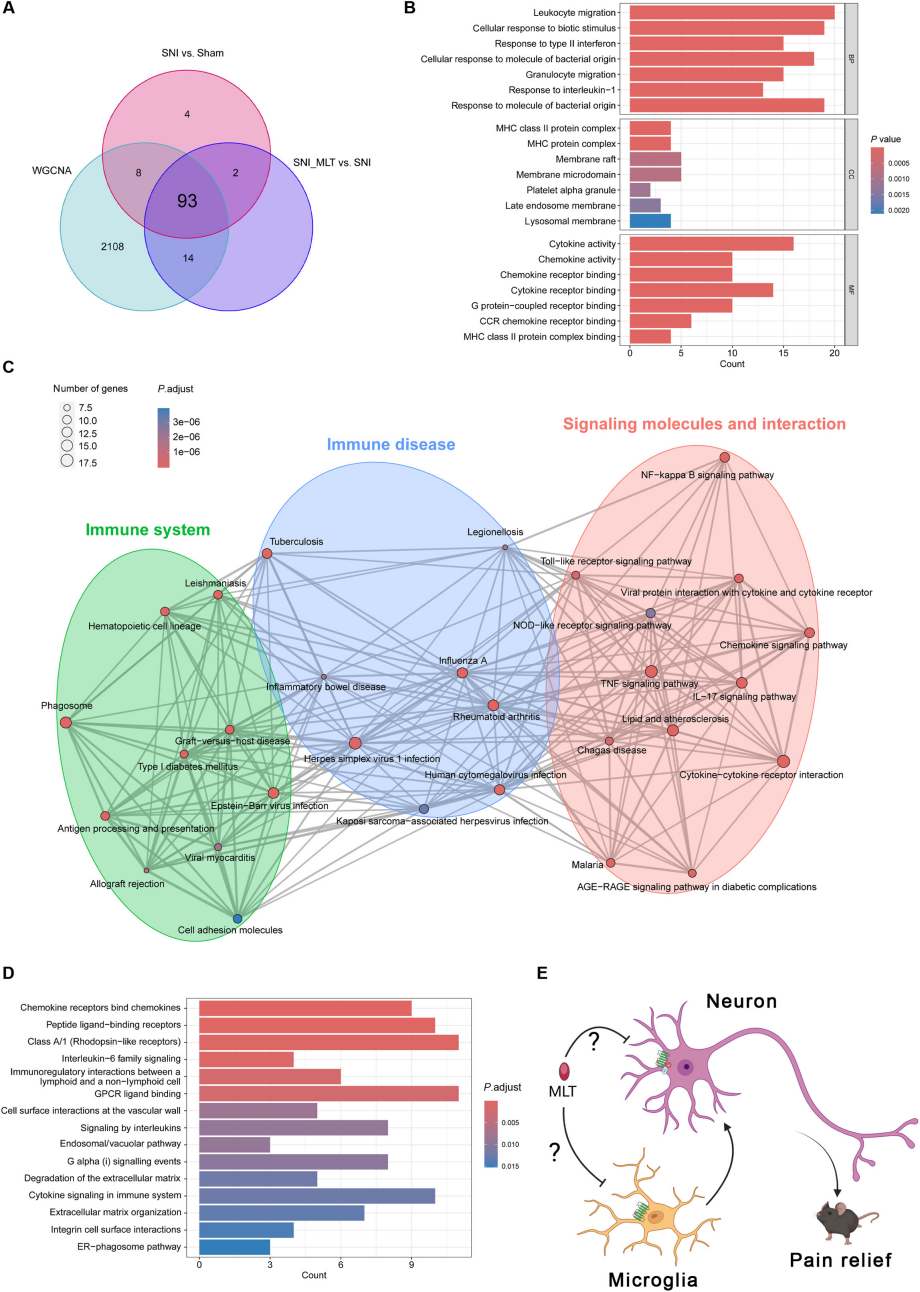
Identification and enrichment analysis of MLT-related genes in SNI mice. (A) Venn diagram illustrating the overlapping genes between DEGs and genes in the green4 modules. (B to D) GO enrichment analysis (B), clustered KEGG pathway enrichment analysis (C), and Reactome pathway enrichment analysis (D) of the overlapping genes in (A). (E) Hypothesized mechanism of MLT’s analgesic effect. Figure 3E was created with BioRender.com, with permission.

To narrow down the genes related to MLT intervention, we identified 93 genes through Venn diagrams by overlapping DEGs with module-related genes (Fig. 3A). Subsequently, we conducted functional enrichment analyses on these 93 genes to gain insights into the potential mechanisms and functions underlying MLT intervention in NP. Gene Ontology (GO) terms highlighted the significant connection of these selected genes in biological processes (BP) such as cellular response to biotic stimulus, response to type II interferon, and response to interleukin-1 (IL-1). The cellular components (CC) results suggested that these genes may function through membrane microdomains and major histocompatibility complex (MHC) protein complexes. Furthermore, molecular function (MF) analysis indicated potential associations with cytokine activation and G protein-coupled receptor (GPCR) binding (Fig. 3B). The Kyoto Encyclopedia of Genes and Genomes (KEGG) enrichment terms discovered the engagement of several signaling pathways pertinent to immune and inflammatory responses, including the immune system, immune disease, and signaling molecules and interactions (Fig. 3C). Reactome enrichment analysis results indicated involvement in GPCR-related pathways [GPCR ligand binding and Gα(i) signaling events] and cytokine signaling in the immune system (Fig. 3D). Based on these results, we hypothesize that MLT may act on both neurons and microglia through GPCR (MT_2_R) to mediate neuroexcitability inhibition and regulate immune inflammation (Fig. 3E).

### MLT reduces the excitability of ACC pyramidal neurons through Gα(i) signaling and suppresses presynaptic excitatory input

**Fig. 4.**
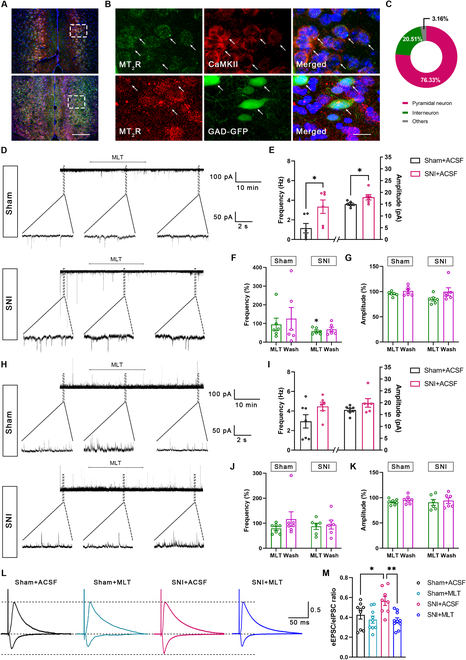
MLT inhibits the frequency of sEPSC of ACC pyramidal cells. (A to C) Immunofluorescence staining for MT_2_R/CaMKII/GAD67-GFP in the ACC. The rectangle areas in (A) are magnified in (B). Scale bars, 200 μm (A) and 10 μm (B). (C) Ratio of MT_2_R-immunoreactivities in CaMKII-immunoreactive cells and GAD67-GFP-labeling cells (*n* = 3). (D) Representative electrophysiological samples showing the sEPSC with bath application of MLT (13.33 μM) in the sham and SNI group. (E) The frequency and amplitude of sEPSC are significantly increased in the SNI mice compared to those in the sham mice. (F) Application of MLT decreases the frequency of sEPSC in the SNI mice but not in the sham mice. (G) Application of MLT has no effect on the amplitude of sEPSC in both the SNI and sham mice. (H) Representative electrophysiological samples showing sIPSCs with bath application of MLT (13.33 μM) in the sham and SNI group. (I) The frequency and amplitude of sIPSC are not altered in the SNI mice compared to those in the sham mice. (J and K) Application of MLT has no effect on the frequency (J) and amplitude (K) of sIPSC in the SNI or sham mice. (L and M) Representative samples (L) and column diagram (M) showing that MLT decreases the ratio of evoked EPSC to IPSC (eEPSC/eIPSC ratio) of the ACC pyramidal cells in SNI mice. *P < 0.05; **P < 0.01.

We first examined the impact of MLT on neuronal excitability. By immunofluorescence staining, 76.33% of MT_2_R-immunoreactive (ir) neurons were found to colabel with CaMKII-ir pyramidal cells, while 20.51% of MT_2_R-ir neurons were colabeled with GAD67-GFP in the ACC (Fig. 4A to C), suggesting that MLT may affect the excitatory and/or inhibitory synaptic transmission and intrinsic properties. We then used the whole-cell patch clamping of ACC pyramidal cells to investigate whether MLT influenced the synaptic transmission. First, the spontaneous excitatory postsynaptic currents (sEPSCs) and spontaneous inhibitory postsynaptic currents (sIPSCs) were recorded to reflect the probability of presynaptic excitatory/inhibitory neurotransmitter discharge and postsynaptic reactions. We observed that both the frequency and amplitude of sEPSCs were raised in the SNI mice, in contrast to those in the sham mice (Fig. 4D and E). Bath application of MLT significantly decreased the frequency of the sEPSCs, but with no effect on their amplitude (Fig. 4F and G). However, both the frequency and the amplitude of sIPSCs were not altered between the SNI and sham mice (Fig. 4H and I), and subsequent bath application of MLT did not change them either (Fig. 4J and K). To evaluate the possible impact of MLT on the excitation–inhibition balance, we recorded the ratio of evoked EPSC to IPSC (E/I ratio) of the same pyramidal cells in the ACC. We observed that SNI enhanced the E/I ratio, which was reduced significantly by bath application of MLT (Fig. 4L and M).

**Fig. 5.**
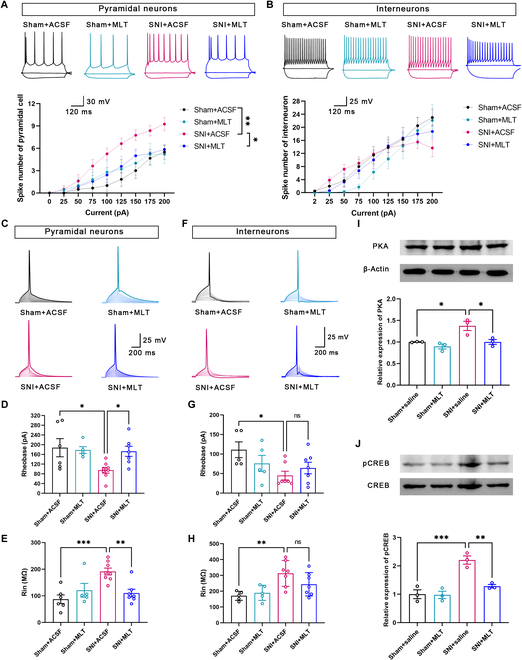
MLT decreases the excitation of ACC pyramidal cells. (A) Application of MLT reduces the spike number of pyramidal neurons in the ACC of SNI mice. (B) Application of MLT has no effect on the spike number of interneurons in the ACC. (C) Representative traces of the first action potential (AP) of pyramidal neurons in the ACC induced by suprathreshold depolarizing current pulse. (D and E) Rheobase current (D) and membrane input resistance (Rin) (E) of ACC pyramidal neurons in sham + ACSF, sham + MLT, SNI + ACSF, and SNI + MLT groups. (F) Representative traces of the first AP of interneurons in ACC induced by suprathreshold depolarizing current pulse. (G and H) Rheobase current (G) and Rin (H) of ACC interneurons in sham + ACSF, sham + MLT, SNI + ACSF, and SNI + MLT groups. (I and J) Samples and column diagram showing that MLT inhibits the increased expression level of PKA and pCREB in SNI mice. *P < 0.05; **P < 0.01; ***P < 0.001.

We then examined whether MLT influenced the intrinsic attributes of ACC pyramidal cells and interneurons. As shown in Fig. 5A, the average number of spikes in ACC pyramidal cells was notably elevated in SNI-induced NP mice when compared to the sham mice. Bath application of MLT reduced the spike number in the SNI group significantly. Additionally, the decreased rheobase current and improved membrane input resistances (Rin) were being observed, which were reversed by MLT application (Fig. 5D and E and Fig. S3). In contrast, MLT application did not change the firing number of action potential, rheobase, or Rin of the interneurons in either SNI or sham mice (Fig. 5B and F to H). Given that MT_2_R is a GPCR signaling through Gα(i) protein [31] (see also Fig. 3D), we performed WB analysis to assess the expression levels of PKA and pCREB, downstream signaling molecules of Gα(i). The results revealed an outstanding increase in the expression levels of PKA and pCREB in the ACC of mice after SNI, and intraperitoneal injection of MLT significantly reduced their expression levels (Fig. 5I and J). Collectively, these findings suggest that MLT reduces the excitatory input to ACC pyramidal cells and inhibits their excitability through Gα(i) signaling.

### MLT inhibits the inflammation and type II interferon response and facilitates the anti-inflammatory polarization of cultured microglia

**Fig. 6.**
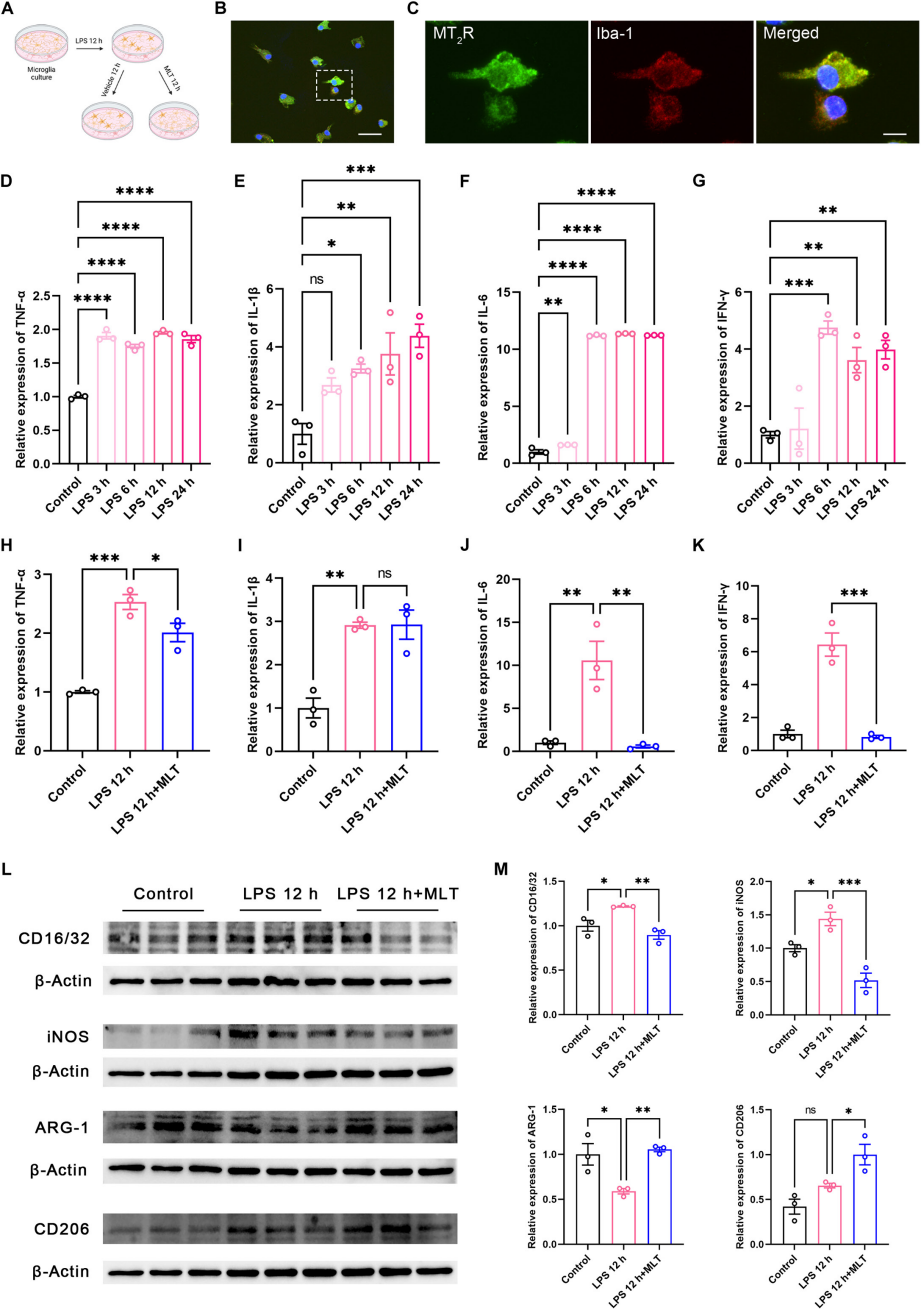
MLT inhibits the M_1_ polarization and promotes M_2_ polarization of primary microglia and reduces LPS-induced elevation of inflammatory factors. (A) Schematic showing the primary microglia culture procedure. (B and C) Immunofluorescence staining for MT_2_R (green), Iba-1 (red), and 4′,6-diamidino-2-phenylindole (DAPI) (blue) in primary microglia. The rectangle area in (B) is enlarged in (C). Scale bars, 100 μm (B) and 10 μm (C). *n* = 3 dishes. (D to G) The levels of TNF-α (D), IL-1β (E), IL-6 (F), and IFN-γ (G) secreted by primary cultured microglia are detected by ELISA at 3, 6, 12, and 24 h after LPS stimulation. (H to K) Effect of MLT on the secretion of TNF-α (H), IL-1β (I), IL-6 (J), and IFN-γ (K) induced by LPS (12-h stimulation) in primary cultured microglia (*n* = 3 dishes). (L and M) Samples (L) and column diagram (M) showing the WB results of the CD16/32, iNOS, ARG-1, and CD206 proteins in primary microglia (*n* = 3 dishes). *P < 0.05; **P < 0.01; ***P < 0.001; ****P < 0.0001. Figure 6A was created with BioRender.com, with permission.

We next inspected the potential influence of MLT on microglia and the ensuing immune-inflammatory response within the ACC. Immunofluorescence staining revealed a high expression of MT_2_R in cultured primary microglia (Fig. 6A to C), consistent with MT_2_R immunofluorescence results in the ACC (Fig. 1J to L). Using enzyme-linked immunosorbent assay (ELISA), the relative expression levels of tumor necrosis factor-α (TNF-α), IL-1β, IL-6, and interferon-γ (IFN-γ) in the culture medium were assessed at 3, 6, 12, or 24 h after lipopolysaccharide (LPS) stimulation. We observed a significant growth in the secretion levels of these inflammatory cytokines at 3 to 24 h after LPS stimulation, as compared to the control group (Fig. 6D to G). The relative secretion of TNF-α, IL-1β, IL-6, and IFN-γ levels at 12 h after LPS stimulation was then utilized to evaluate the MLT intervention effect. The application of MLT markedly inhibited the relative secretion levels of TNF-α, IL-6, and IFN-γ, without affecting the secretion level of IL-1β (Fig. 6H to K).

Prior research has demonstrated that microglia exhibit high sensitivity to microenvironmental signals, leading to either M_1_-like (proinflammatory phenotype) or M_2_-like (anti-inflammatory phenotype) polarization [32]. This prompted our hypothesis that MLT might regulate immune inflammation by modulating microglial polarization. To assess this, we performed WB analysis to examine the expression levels of 4 microglial polarization markers: CD16/32 and inducible nitric oxide synthase (iNOS) for the M_1_ phenotype, and arginase-1 (ARG-1) and CD206 for the M_2_ phenotype. Our findings exposed that LPS stimulation increased the expression of CD16/32 and iNOS, while MLT attenuated these elevated expression noticeably. Conversely, LPS down-regulated the expression intensity of ARG-1 and CD206, and MLT increased these reduced expression considerably (Fig. 6L and M). These results suggest that MLT inhibits inflammation and the type II interferon response mediated by M_1_-like microglia while facilitating the shift of microglia to the M_2_ phenotype.

### MLT inhibits inflammation and type II interferon response through cGAS-STING signaling pathway in the ACC

**Fig. 7.**
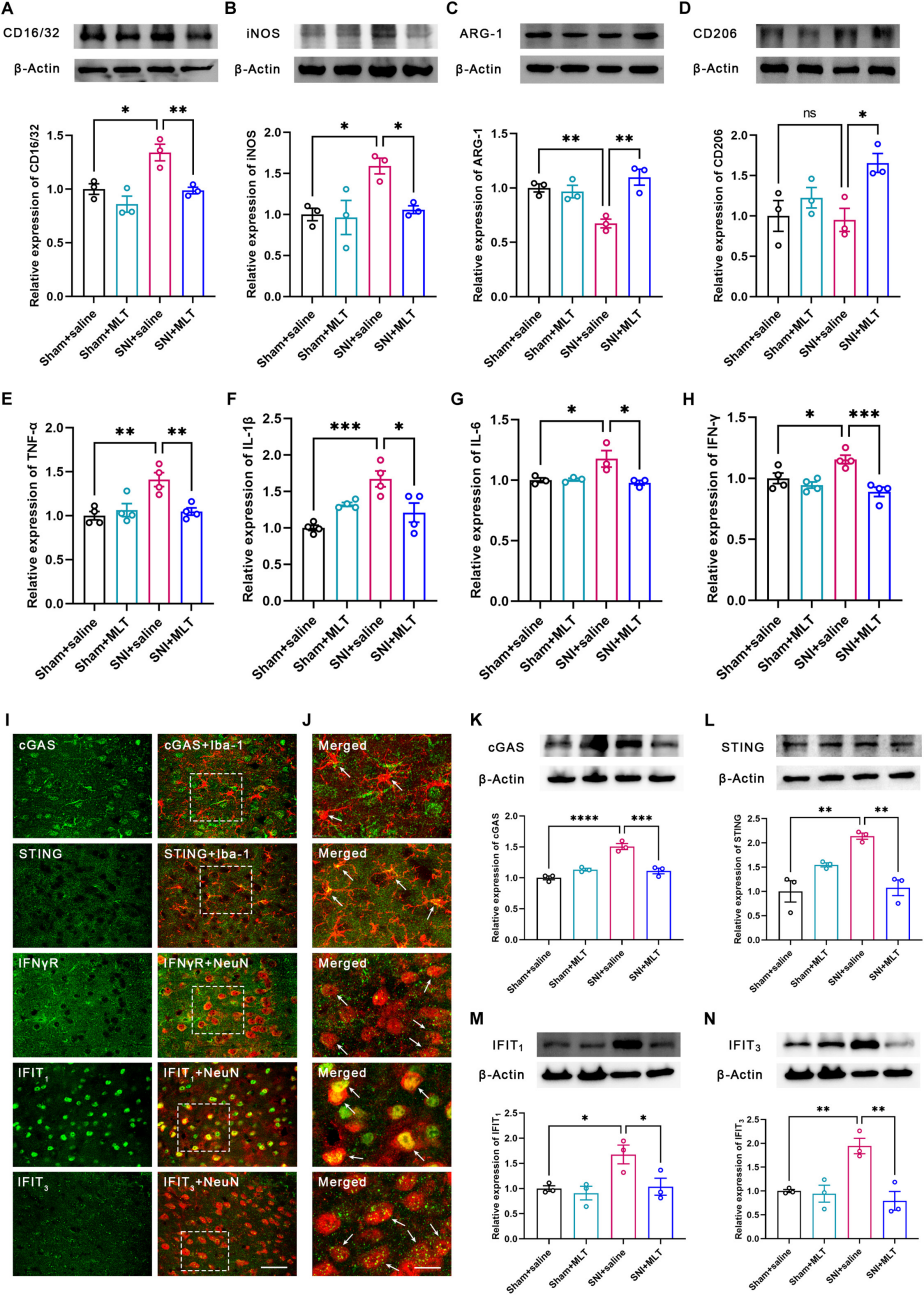
MLT inhibits inflammation and type II interferon response in the ACC through cGAS-STING signaling pathway. (A to D) Samples and column diagram showing the WB results of CD16/32, iNOS, ARG-1, and CD206 proteins in the mice ACC (*n* = 3). (E to H) SNI increases the level of TNF-α (E), IL-1β (F), IL-6 (G), and IFN-γ (H) in the ACC, and MLT reduces the level of these cytokines apparently [*n* = 4 in (E), (F), and (H); *n* = 3 in (G)]. (I and J) Immunofluorescence staining results for cGAS/Iba-1, STING/Iba-1, IFNγR/NeuN, IFIT_1_/NeuN, and IFIT_3_/NeuN in the ACC. The rectangle areas in (I) are enlarged in (J). Scale bars, 100 μm (I) and 10 μm (J). (K to N) Samples and column diagram showing the WB results of the cGAS (K), STING (L), IFIT_1_ (M), and IFIT_3_ (N) proteins in the ACC. *P < 0.05; **P < 0.01; ***P < 0.001; ****P < 0.0001.

To reinforce our in vivo results with further evidence, we utilized WB analysis and ELISA to assess the expression levels of 4 microglial polarization marker molecules in the ACC. We observed that SNI treatment significantly elevated the expression levels of CD16/32 and iNOS in the ACC; however, these levels were reduced by MLT intraperitoneal administration (Fig. 7A and B). Conversely, although the expression levels of ARG-1 but CD206 were reduced by SNI treatment, MLT intraperitoneal administration markedly enhanced their expression (Fig. 7C and D). Consistently, we verified that the expression levels of proinflammatory cytokines, including TNF-α, IL-1β, IL-6, and IFN-γ, were substantially elevated in the ACC by SNI treatment. Importantly, MLT administration significantly mitigated the expression levels of these proinflammatory mediators (Fig. 7E to H).

Prior research has suggested that the stimulator of interferon genes (STING) releases proinflammatory cytokines, contributing to neuroinflammation that exacerbates chronic pain [33–35]. We then tested whether MLT diminishes inflammation and type II interferon response via the cyclic guanosine monophosphate–adenosine monophosphate synthase (cGAS)-STING signaling pathway. We first investigated the cell types expressing cGAS, STING, IFNγR, IFIT_1_ (interferon-induced proteins with tetratricopeptide repeats 1), and IFIT_3_ in the ACC and observed an abundant expression of cGAS and STING in microglia, while IFNγR, IFIT_1_, and IFIT_3_ were predominantly expressed in neurons (Fig. 7I and J). Subsequently, we conducted WB analysis and found that the expression levels of cGAS, STING, IFIT_1_, and IFIT_3_ were significantly elevated in SNI mice, and MLT application led to a substantial reduction in their expression levels (Fig. 7K to N). These findings strongly support that MLT should suppress inflammation and type II interferon response through the cGAS-STING signaling pathway.

## Discussion

**Fig. 8.**
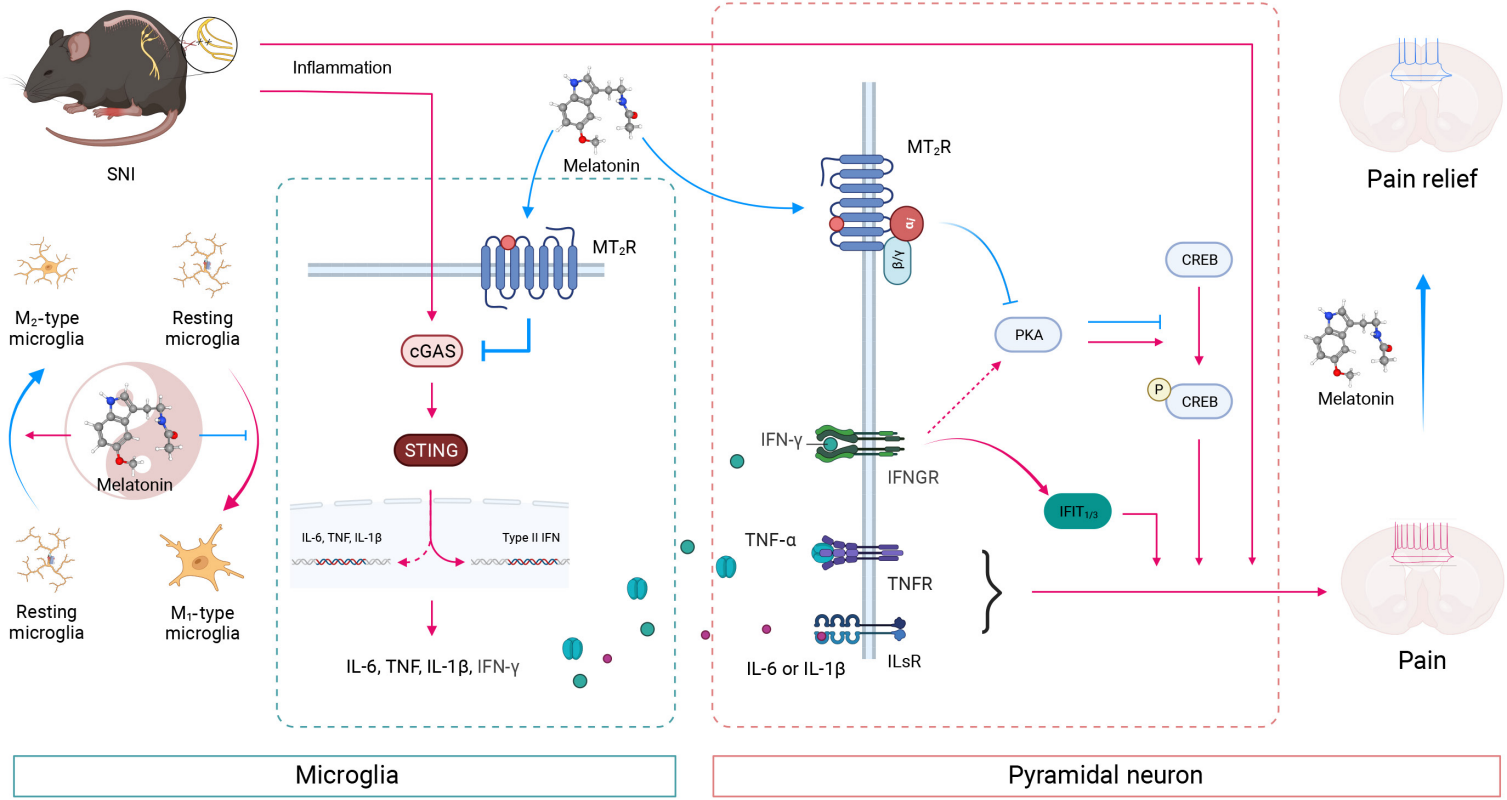
Schematic model of MLT’s analgesic effects on SNI-induced NP. MLT, through MT_2_R, inhibits the excitability of pyramidal cells in the ACC by activating the Gα(i) signal. Concurrently, MLT inhibits M_1_ polarization of microglia while promoting M_2_ polarization, thereby mitigating the inflammatory response and type II interferon response in the ACC. Image was created with BioRender.com, with permission.

Within this research, we used the SNI model to induce NP in mice and demonstrated that both intraperitoneal injection and ACC microinjection of MLT effectively mitigate NP in a dose-dependent manner. The analgesic properties of MLT are specifically mediated through MT_2_R, a GPCR predominantly expressed in ACC neurons and microglia. Mechanistically, MLT utilizes the activation of the Gα(i) signaling pathway to deliver its analgesic benefits through MT_2_R, leading to the inhibition of pyramidal neuron excitability in the ACC. Simultaneously, MLT impedes the M_1_ polarization of microglia and promotes their shift toward the M_2_ phenotype, thereby reducing inflammatory responses and type II interferon reactions in the ACC (Fig. 8).

MLT, often referred to as the pineal hormone, is an indole-like neuroendocrine bioactive substances produced by the pineal gland. Its primary physiological role involves the regulation of the human biological rhythm, with a particular emphasis on the circadian rhythm [36–38]. Beyond its circadian functions, MLT also demonstrates various physiological effects, including antioxidative capabilities [39], immune modulation [40,41], influences on neuroendocrine function and aging [42], and analgesic properties [43,44]. In humans and other mammals, the primary mediators of MLT’s biological functions are MT_1_R and MT_2_R, which exhibit substantial amino acid homology and cooperatively regulate circadian rhythms and immune responses within the central nervous system (CNS). In the present study, to eliminate the possible influence of rhythm variations on pain perception, we chose to conduct behavioral tests for all groups during the same period of the day. Notably, our study reveals a considerable dose-dependent alleviation of NP through intraperitoneal or ACC administration of MLT, and this analgesic effect is specifically attributed to MT_2_R. Previous studies have shown that *N*-{2-([3-bromophenyl]-4-fluorophenylamino)ethyl} acetamide (UCM924), a selective agonist of MT_2_R, exerts analgesic effects in MT_1_^−/−^ mice but not in MT_2_^−/−^ and MT_1_^−/−^/MT_2_^−/−^ mice [45]. Our finding aligns with previous rodent studies indicating that MT_2_R, rather than MT_1_R, plays a crucial role in pain modulation [26,27,45].

The ACC has emerged as a pivotal region in various brain functions, encompassing pain processing, fear memory, and social behavior [6,10,12,14,46]. A growing body of research implicates aberrant excitability in ACC neurons and hyperactivity of glial cells, particularly microglia, in pain hypersensitivity [47–50]. Understanding the intricate interplay between neurons and microglia offers insights into mechanisms amplifying pain signals associated with NP, offering potential avenues for the development of novel therapeutic interventions for chronic pain. Our study revealed that approximately 73.62% of MT_2_R-ir signals were localized to neuronal membranes, with 23.44% observed in microglial membranes. Furthermore, MLT orchestrates the regulation of immune-inflammatory responses through GPCR signaling and type II interferon responses, as revealed by RNA-sequencing (RNA-seq) analysis. These findings suggest that MLT’s analgesic effects may directly influence neuronal excitability and modulate microglial activity through immune-inflammatory regulation. Our investigation explores MLT’s mechanism from the perspectives of both neurons and microglia. Results indicated that MLT suppressed excitatory input to ACC neurons and inhibited pyramidal neuron excitability through Gα(i) signaling. While we observed MT_2_R expression in interneurons, MLT’s impact on interneuronal electrophysiological characteristics was not evident. A previous study has shown that FOS protein expression is primarily localized to pyramidal cells within the ACC of rats 1 month following Complete Freund’s Adjuvant (CFA) injection, with no significant expression observed in interneurons [51]. Our previous study also reveals that in mice, 1 week after NP, the FOS expression and firing rate of pyramidal neurons in the ACC are elevated, whereas those of interneurons remain unchanged [10]. Thus, solely focusing on the perspective of ACC, these findings align with our present electrophysiological outcomes, suggesting that the interneurons located within the ACC may not be implicated in the modulation of chronic pain. However, considering that optogenetic activation [13] or chemogenetic inhibition [51] of interneurons in the ACC has been demonstrated to modulate mechanical pain responses or anxiety-like behaviors induced by inflammatory pain, respectively, it seems improbable that interneurons play no significant role in the regulation of nociception. Therefore, we hypothesize that the involvement of ACC interneurons in the regulation of chronic pain may be more intricate and multifaceted than previously envisioned. A more reasonable hypothesis may be that interneurons play a phasic but not tonic inhibitory role in the global activity of ACC, thus regulating nociception.

Additionally, Lopez-Canul et al. [52] find that UCM924 notably alleviates pain behaviors in rats after L_5_–L_6_ spinal nerve ligation and SNI. This mechanism may be closely related to the decreased activity of pronociceptive ON cells and the enhanced activity of antinociceptive OFF cells in the periaqueductal gray (PAG) induced by UCM924. Based on the results of our study, we propose that the analgesic effect of MLT is associated with the expression and distribution of MT_2_R in the brain, and ACC is a critical but not the only brain region for MLT to exert its analgesic effect. Furthermore, Posa et al. [53] reveal that the antinociceptive effect of UCM924 in the rostral ventromedial medulla (RVM) is blocked by mu opioid receptor (MOR), but not delta opioid receptor (DOR), antagonism. Given the key role of MOR in the descending analgesic circuit, and the fact that ACC projecting to the PAG [54,55], we speculate that MLT may affect the activity of ACC projecting pyramidal neurons and also in turn regulate the descending analgesic circuit in the PAG and/or RVM, thereby producing an analgesic effect.

In recent investigations on microglia, it has been demonstrated that reactive microglia contribute to the sustained excitability of ACC neurons in reply to chronic nicotine exposure. Notably, pharmacological interventions specifically targeting microglia, including minocycline or liposome clodronate, have been shown to effectively alleviate nicotine-induced allodynia [50]. Furthermore, investigations into the inflammatory response triggered by spinal cord injury have identified distinct microglial subgroups, categorized as either classically activated (M_1_ phenotype) or alternatively activated (M_2_ phenotype) [29,56]. Analogous to macrophages, the M_1_ phenotype exhibits heightened expression of TNF-α, IL-1β, IL-6, and IFN-γ, which are implicated in NP or osteoarthritis progression. Conversely, the M_2_ phenotype displays augmented anti-inflammatory properties [56,57]. In our current study, we discovered that MLT decreased the release of inflammatory factors like TNF-α, IL-1β, IL-6, and IFN-γ, which are typically orchestrated by M_1_-like microglia. Simultaneously, it promoted the polarization of microglia toward the M_2_ phenotype. This suggests that MLT achieves a reduction in inflammation by effectively inhibiting the polarization of microglia toward the inflammatory M_1_ phenotype.

The cGAS-STING pathway has taken center stage as a key regulator of inflammatory disorders, such as infection [58] and brain damage [59]. A recent investigation has revealed that the microglial M_1_ polarization triggered by LPS occurs simultaneously with the triggering of the cGAS-STING pathway. Especially, blocking cGAS-STING signaling reduces the LPS-induced M_1_ polarization of microglia. In vivo experiments further demonstrate that treatment with the antagonists of cGAS and STING mitigates microglial M_1_ polarization, thereby alleviating SNI-induced mechanical allodynia [60]. We observed that both the cGAS and STING protein were mainly noted in microglia and their expression levels increased in the ACC after SNI, and MLT reduced their expression markedly, suggesting that MLT’s inflammatory regulatory effect is mediated through the inhibition of cGAS-STING signaling pathway. Previous research has indicated that the cGAS-STING signaling pathway can trigger the canonical IRF3 phosphorylation pathway, which leads to the secretion of type I IFNs (IFN-α and IFN-β) as well as enhanced expression of IFN-stimulated genes (ISGs) [61], and a noncanonical nuclear factor κB (NF-κB) pathway for the expression of type II IFN (IFN-γ) and ISG [61–63]. We also found that the receptor for IFN-γ is predominantly expressed in neurons of the ACC, and MLT significantly reduced the expression level of 2 ISGs, IFIT_1_ and IFIT_3_, which were up-regulated via SNI. From the RNA-seq and experimental results, it could be seen that MLT reduced the IFN-γ response caused by SNI, so the changes in the expression of IFIT_1_ and IFIT_3_ by MLT might be related to its modulation of IFN-γ. IFN-γ is recognized for its ability to amplify microglia’s proinflammatory reactions in cases of pain, and it aids in synapse elimination and nitric oxide liberation, ultimately disrupting synaptic transmission and cognitive functions [64–67]. IFIT_1_ and IFIT_3_, members of the ISG family, play crucial roles in antiviral immune response, innate immunity mechanisms, and the inflammatory response [68,69]. Collectively, the above results suggest the potential mechanism by which MLT indirectly influences the activity of ACC pyramidal neurons via the immune-inflammatory pathway.

In conclusion, our research sheds light on the dual effects of MLT on neurons and microglia, enhancing our comprehensive understanding of its analgesic mechanisms in NP. The findings uncover a novel neuro-immune regulatory mechanism mediated by MLT in the ACC, which is pivotal in regulating NP in mice. This study also represents an important step forward in informing the development of innovative therapeutic approaches for chronic pain and its associated symptoms.

## Materials and Methods

### Animals

Male mice (6 to 8 weeks) from 2 strains, C57BL/6J and GAD67-GFP (C57 genetic background), were utilized. Unlimited access to food and water as well as environmentally controlled conditions (12-h light/dark cycle, stable temperature of 22 ± 1 °C, and humidity maintained at 60 ± 5%) were provided for all animals. It is crucial to emphasize that strict ethical guidelines for pain research were followed, and the experimental protocols applied in the present study were approved by the Ethics Committee on Animal Application for Research and Education of the Fourth Military Medical University and the Administrative Panel on Laboratory Animal Care at the National Institute of Biological Sciences.

### Drugs

MLT (CAS No.: 73-31-4, MedChemExpress) was dissolved in a solution comprising dimethyl sulfoxide (D2650, Sigma) and 0.9% saline, and then administered intraperitoneally at varying doses (0.1, 0.3, 1, 3, 10, and 1.114 mg/kg). 8-M-PDOT (CAS No.: 134865-70-6, MedChemExpress), 4-P-PDOT (CAS No.: 134865-74-0, MedChemExpress), and Ramelteon (CAS No.: 196597-26-9, MedChemExpress) were dissolved in a solution of 0.9% saline.

### Spared nerve injury

The surgical procedures were conducted in compliance with previously published protocols [5]. To put it briefly, mice were anesthetized using 2% isoflurane. The skin and a segment of the biceps femoris muscle in the left thigh were carefully incised to expose 3 terminal branches of the sciatic nerve. Specifically, the tibial nerve and the common peroneal nerve were securely tied off using 6-0 silk sutures and then severed just below the ligation point. Following this ligation and transection, the stump of nerve fiber was gently returned to the place where it should be, and the incision in the muscle and skin was sutured in 2 distinct layers to ensure proper closure. In contrast, the sham mice underwent a surgical procedure solely to expose the left sciatic nerve branches, without inflicting any injury to the nerves.

### Measurement of the mechanical PWMT

PWMT of the hind paw with surgical or sham operation was assessed using a comprehensive series of von Frey filaments (Stoelting Company, Wood Dale, USA) during the same period of the day (from 14:00 to 16:00), following established methodologies from previous studies [29,70]. In brief, mice were placed in customized metal mesh grids (7 × 7 × 10 cm^3^) and enclosed in a transparent engineering plastic cover for a 30-min acclimatization period before PWMT tests. The assessments were conducted at baseline (day 0), days 1 to 6, and following intraperitoneal injection of MLT after SNI/saline treatment. After the mice became accustomed to the environment, a diverse range of filaments with graduated strengths (0.008, 0.02, 0.04, 0.16, 0.4, 0.6, 1, and 1.4 g), equivalent to escalating forces (0.078, 0.196, 0.392, 1.568, 3.92, 5.88, 9.8, and 13.72 mN), were vertically applied to determine the PWMT. This threshold was defined as the minimum bending force required to trigger a withdrawal response in 3 of 5 stimulation attempts.

### Primary culture of microglia

Primary microglial, according to previous studies, were extracted from newborn C57BL/6J mice [71,72]. Briefly, newborn mice were cleansed with 75% alcohol; subsequently, their entire brains were extracted and finely chopped in precooled phosphate-buffered saline (PBS). Later, the cortical tissue was subjected to a 20-min digestion with trypsin (0.25%). After centrifugation and resuspension, the samples were digested with deoxyribonuclease I at 37 °C to obtain a single-cell suspension. Subsequently, these individual cells were seeded onto poly-d-lysine-coated flasks and incubated for 14 d. To isolate microglial cells from the mixed glial cultures, the flasks were shaken at 180 rpm for 3 h.

### Immunofluorescent staining

Mice were deeply anesthetized with 2% isoflurane and then perfused immediately with 50 ml of 0.9% saline. When 0.9% saline is exhausted, 100 ml of 0.1 M phosphate buffer (PB) containing 4% paraformaldehyde was followed quickly. After perfusion ended, the brains were extracted gently and immersed rapidly in 0.1 M PB mixed with 30% sucrose at 4 °C overnight. The next day, the brains were sliced into consecutive 30-μm-thick frontal sections with a freezing microtome (Kryostat 1720; Leitz, Mannheim, Germany). These sections were systematically collected and washed with PBS (0.01 M, pH 7.4). Subsequently, the sections were soaked in PBS mixed with Triton X-100 (0.3%) and normal goat serum (NGS; 1%) for half an hour.

For MT_2_R/NeuN/Iba-1/GFAP (glial fibrillary acidic protein) immunofluorescence staining, the selected brain sections were incubated with rabbit anti-MT_2_R (#AMR-032, 1:200; Alomone, Jerusalem Biopark, Israel), mouse anti-NeuN (1:500; MAB377, MerckMillipore), goat anti-Iba-1 (1:200; ab5076, Abcam, Cambridge, UK), and mouse anti-GFAP (1:4,000; MAB3402, MerckMillipore, Massachusetts, USA) antibodies. For MT_2_R/CaMKII, sections were incubated with mouse anti-CaMKII (1:100, ab22609, Abcam) and rabbit anti-MT_2_R antibodies. For cGAS/Iba-1 and STING/Iba-1, sections were incubated with goat anti-Iba-1 and rabbit anti-cGAS (1:150; NBP3-16666, Novus, Colorado, USA) and rabbit anti-STING (1:50; ab288157, Abcam) antibodies. For IFNγR/IFIT_1_/IFIT_3_/NeuN, sections were incubated with mouse anti-NeuN and rabbit anti-IFN gamma receptor beta/AF-1 (1:500, ab224197, Abcam), rabbit anti-IFIT_1_ (1:200; abs116420, Absin, Shanghai, China), and rabbit anti-IFIT_3_ (1:200; abs116420, Absin, Shanghai, China) antibodies. All antibodies were diluted in PBS containing 5% NGS, 0.3% Triton X-100, 0.05% NaN_3_, and 0.25% carrageenan (PBS-NGS, pH 7.4) overnight at 4 °C.

After incubation, the sections were rinsed and exposed to fluorophore-conjugated secondary antibodies (1:200, Invitrogen, ThermoFisher, CA, USA) at room temperature for 4 to 6 h. In the end, all sections were mounted on glass slides and inspected utilizing a laser scanning confocal microscope (FV1000, Olympus, Japan) or a slide scanner (Slideview VS200, Olympus) equipped with appropriate filters.

### RNA-seq and bioinformatics analysis

We extracted the total RNA from the ACC brain regions of mice in the sham + saline group, SNI + saline group, and SNI + MLT group, strictly following the instructions provided by the Trizol reagent kit (Invitrogen). First, the quality of the extracted RNA was evaluated using an Agilent 2100 Bioanalyzer (Agilent Technologies, Palo Alto, CA, USA) and further verified through ribonuclease (RNase)-free agarose gel electrophoresis. Subsequently, eukaryotic mRNA was enriched utilizing oligo(dT) beads. The enriched mRNA was then fragmented using a specific buffer and reverse-transcribed into cDNA with the assistance of random primers. The synthesis of the second strand of cDNA was carried out using DNA polymerase I, RNase H, deoxynucleotide triphosphate (dNTP), and buffer. Afterward, the cDNA fragments were purified using the QiaQuick polymerase chain reaction (PCR) extraction kit (Qiagen, Venlo, The Netherlands). These fragments then underwent end repair, poly(A) tail addition, and ligation to Illumina sequencing adapters. The ligation products were size-selected via agarose gel electrophoresis, amplified using PCR, and finally sequenced on the Illumina Novaseq6000 by Gene Denovo Biotechnology Co. (Guangzhou, China).

To identify DEGs, the “limma” R package was applied with a significance threshold of *P* < 0.05 and |log_2_ fold change| > 1.2. Visualization was achieved using the renowned R packages “ggplot2” and “pheatmap” [73]. WGCNA with the corresponding R package was performed to construct scale-free coexpression networks specific to MLT intervention [74]. To begin with, hierarchical clustering analysis helped filter out discrete cases. An optimal soft power b was then chosen to create the weighted adjacency matrix, later transformed into a topological overlap matrix (TOM). This TOM included module assignments represented by colors and module eigenvectors (MEs). Moreover, Pearson correlation coefficients were analyzed to determine the relationship between ME and MLT intervention. Hub genes were selected from the module most relevant to MLT intervention in WGCNA, based on criteria of gene significance (GS) > 0.25 and module membership (MM) > 0.7. Genes related to both SNI and MLT were identified by intersecting hub genes and DEGs, visualized using a Venn diagram.

To further investigate the biological mechanisms associated with the hub DEGs obtained by intersection, the R packages “clusterProfiler” and “org.Mm.eg.db” were then utilized to perform GO, KEGG, and Reactome enrichment analyses with *q* value < 0.05 as the threshold for significant enrichment [74]. The raw sequence data reported in this paper have been deposited in the Genome Sequence Archive in National Genomics Data Center, China National Center for Bioinformation/Beijing Institute of Genomics, Chinese Academy of Sciences (GSA: CRA018099) that are publicly accessible at https://ngdc.cncb.ac.cn/gsa.

### Western blotting

Mice were euthanized under deep anesthesia using 2% isoflurane, and the entire brains were swiftly extracted. The ACC was extracted and uniformly mixed using an automated rapid sample homogenizer (Jingxin industry, Shanghai, China) in sodium dodecyl sulfate (SDS) sample buffer. Subsequently, all the samples were heated with water bath at 100 °C for a duration of 10 min, loaded onto gels, and separated via electrophoresis on 10% SDS-polyacrylamide gels utilizing standard Laemmli solutions (Bio-Rad Laboratories, CA, USA). The proteins were then blotted onto a polyvinylidene difluoride membrane (Immobilon-P, Millipore, Hayward, CA, USA). Next, the membranes were immersed in a blocking solution for 1 h, followed by overnight incubation under gentle agitation with primary antibodies: rabbit anti-MT_1_R (#AMR-031, 1:800; Alomone, Jerusalem Biopark, Israel), rabbit anti-MT_2_R (#AMR-032, 1:800; Alomone), rabbit anti-PKA (1:1,000; ab75991, Abcam), rabbit anti-pCREB (1:1,000; 9198, Cell Signaling Technology, USA), rabbit anti-CREB (1:1,000; 9197, Cell Signaling Technology), mouse anti-CD16/32 (1:800; 553141, BD Pharmingen, USA), mouse anti-iNOS (1:1,000; ab49999, Abcam), mouse anti-ARG-1 (1:1,000; 93668S, Cell Signaling Technology), rabbit anti-cGAS (1:500; NBP3-16666, Novus, Colorado, USA), rabbit anti-STING (1:500; ab288157, Abcam), or rabbit anti-IFIT_1_ (1:800; ab236256, Abcam) and rabbit anti-IFIT_3_ (1:800; abs116420, Absin, Shanghai, China). After that, we applied horseradish peroxidase (HRP)-conjugated secondary antibodies to detect primary antibodies, specifically anti-rabbit (1:5,000; ZB-2301, ZSGB-BIO, Beijing, China) or anti-mouse (1:5,000; ZB-2305, ZSGB-BIO). Visualization of all reactions was achieved through the enhanced chemiluminescence (ECL) detection technique, and the intensities of the protein bands were quantified using Labworks Software (Ultra-Violet Products, UK).

### ELISA measurement of IL-6, IL-1β, TNF-α, and IFN-γ in primary microglia and ACC tissue

Primary microglia and ACC samples underwent homogenization in physiological saline via an automated rapid sample homogenizer, followed by centrifugation at 1,000*g* for 10 min in a low-temperature high-speed centrifuge maintained at −4 °C. The processed samples were then aliquoted and preserved at −80 °C for future examination.

For cytokine quantification, we employed mouse IL-6, IL-1β, TNF-α, and IFN-γ Valukine ELISA Kits from BioTechne (USA), adhering strictly to the manufacturer’s guidelines. The cytokine levels were determined using an EnSpire Multimode Plate Reader provided by PerkinElmer (USA).

### Whole-cell patch-clamp recordings

The experimental procedures were carried out in accordance with a predefined protocol [28,29]. Concisely, mice were anesthetized and followed by sacrifice through decapitation. Using a vibrating microtome (Leica VT 1200s, Heidelberger, Nussloch, Germany), transverse slices (300 μm thick) encompassing the ACC were precisely cut at temperatures ranging from 0 to 4 °C. This was done in oxygenated artificial cerebrospinal fluid (ACSF; 95% O_2_ and 5% CO_2_) composed of 124 mM NaCl, 25 mM NaHCO_3_, 10 mM glucose, 2.5 mM KCl, 1 mM NaH_2_PO_4_, 2 mM CaCl_2_, and 1 mM MgSO_4_. After cutting, the slices were moved to a recovery chamber maintained at room temperature and filled with oxygenated ACSF. The slices were then incubated at room temperature for 1 h prior to patch clamp recording.

Neurons were visualized with a microscope featuring infrared differential interference contrast or fluorescent optics video microscopy (Olympus BX51WI). Recordings were made in voltage-clamp or current-clamp modes using an Axon 700B amplifier (Molecular Devices, USA), and data were captured via Clampex software (Molecular Devices). In current-clamp mode, recording pipettes (3 to 5 MΩ) were filled with an internal solution (adjusted to pH 7.2 with KOH, 290 mOsmol) containing 124 mM K-gluconate, 10 mM HEPES, 10 mM phosphocreatine disodium, 5 mM NaCl, 2 mM MgATP, 1 mM MgCl_2_, 0.2 mM EGTA, and 0.1 mM Na_3_GTP. The firing patterns of ACC neurons were documented by recording action potential trains triggered by intracellular injection of gradient current (−100 to 200 pA, interval 25 pA, 400 ms). The first (suprathreshold) action potential was induced by intracellular injection of depolarizing currents, starting from 0 pA and incrementing by 5 pA (30 ms). For EPSC and IPSC recording, the pipette solution (adjusted to pH 7.2 with CsOH, 290 mOsmol) comprised 112 mM Cs-gluconate, 10 mM HEPES, 5 mM TEA-Cl, 5 mM QX-314, 3.7 mM NaCl, 2 mM MgATP, 0.3 mM Na_3_GTP, and 0.2 mM EGTA. EPSCs were captured with the cell membrane potential maintained at −70 mV, while IPSCs were recorded at 0 mV. Except for γ-aminobutyric acid (GABA)-related current recordings, all experiments were conducted with the application of picrotoxin (Sigma-Aldrich) in the bath (100 μM). A bipolar stimulation electrode linked to an isolation current stimulator (Natus Medical Incorporated, Canada) was utilized for eEPSC/eIPSC experiments at 20-μA intensity. The stimulus-generated eEPSC amplitude to eIPSC amplitude ratio was computed as the E/I ratio. The initial access resistance was between 15 and 30 MΩ and continuously monitored. It was worth noting that data were discarded if the access resistance varied by >15% during the present experiment. The data were filtered at 1 kHz and digitized at 10 kHz. All whole-cell patch data analyses were conducted using the Mini Analysis Program (Synaptosoft, USA) and Clampfit 10.2.

### Statistical analysis

All experiments and data analyses in the present study were conducted in a strictly blinded manner. For statistical evaluations and graph creation, we utilized GraphPad Prism (version 9.5.1). Additionally, bioinformatic assessments and visualizations were performed with R (version 4.3.2). Regarding the calculation of ED_50_ concentration of MLT, the administered doses of MLT, whether through intraperitoneal injection or microinjection, were converted to logarithmic scales. Subsequently, a nonlinear regression model was employed to generate a dose–effect curve, which was then leveraged to ascertain the ED_50_ value. The ED_50_ signifies the median effective dose necessary to elicit an analgesic response. To determine statistical significance, we used paired or unpaired *t* tests, 1-way or 2-way analysis of variance (ANOVA), and 2-way repeated-measures ANOVA, with subsequent Holm–Sidak test for post hoc comparisons. Experimental data are shown as mean ± SEM, with statistical significance set at *P* < 0.05.

## Data Availability

The datasets used and/or analyzed during the current study are available from the corresponding author on reasonable request.

## References

[1] Balanaser M, Carley M, Baron R, Finnerup NB, Moore RA, Rowbotham MC, Chaparro LE, Gilron I. Combination pharmacotherapy for the treatment of neuropathic pain in adults: Systematic review and meta-analysis. Pain. 2023;164(2):230–251. 35588148 10.1097/j.pain.0000000000002688

[2] Tesfaye S, Sloan G, Petrie J, White D, Bradburn M, Julious S, Rajbhandari S, Sharma S, Rayman G, Gouni R, et al. Comparison of amitriptyline supplemented with pregabalin, pregabalin supplemented with amitriptyline, and duloxetine supplemented with pregabalin for the treatment of diabetic peripheral neuropathic pain (OPTION-DM): A multicentre, double-blind, randomised crossover trial. Lancet. 2022;400(10353):680–690. 36007534 10.1016/S0140-6736(22)01472-6 PMC9418415

[3] Ferreira GE, Abdel-Shaheed C, Underwood M, Finnerup NB, Day RO, McLachlan A, Eldabe S, Zadro JR, Maher CG. Efficacy, safety, and tolerability of antidepressants for pain in adults: Overview of systematic reviews. BMJ. 2023;380: Article e072415. 36725015 10.1136/bmj-2022-072415 PMC9887507

[4] Acioglu C, Heary RF, Elkabes S. Roles of neuronal toll-like receptors in neuropathic pain and central nervous system injuries and diseases. Brain Behav Immun. 2022;102:163–178. 35176442 10.1016/j.bbi.2022.02.016

[5] Campbell JN, Meyer RA. Mechanisms of neuropathic pain. Neuron. 2006;52(1):77–92. 17015228 10.1016/j.neuron.2006.09.021 PMC1810425

[6] Bliss TV, Collingridge GL, Kaang BK, Zhuo M. Synaptic plasticity in the anterior cingulate cortex in acute and chronic pain. Nat Rev Neurosci. 2016;17(8):485–496. 27307118 10.1038/nrn.2016.68

[7] Chen QY, Li XH, Zhuo M. NMDA receptors and synaptic plasticity in the anterior cingulate cortex. Neuropharmacology. 2021;197: Article 108749. 10.1016/j.neuropharm.2021.108749 34364898

[8] Li ZZ, Han WJ, Sun ZC, Chen Y, Sun JY, Cai GH, Liu WN, Wang TZ, Xie YD, Mao HH, et al. Extracellular matrix protein laminin β1 regulates pain sensitivity and anxiodepression-like behaviors in mice. J Clin Invest. 2021;131(15): Article e146323. 34156983 10.1172/JCI146323 PMC8321574

[9] Koga K, Descalzi G, Chen T, Ko HG, Lu J, Li S, Son J, Kim T, Kwak C, Huganir RL, et al. Coexistence of two forms of LTP in ACC provides a synaptic mechanism for the interactions between anxiety and chronic pain. Neuron. 2015;85(2):377–389. 25556835 10.1016/j.neuron.2014.12.021 PMC4364605

[10] Zhu DY, Cao TT, Fan HW, Zhang MZ, Duan HK, Li J, Zhang XJ, Li YQ, Wang P, Chen T. The increased in vivo firing of pyramidal cells but not interneurons in the anterior cingulate cortex after neuropathic pain. Mol Brain. 2022;15(1):12. 35093140 10.1186/s13041-022-00897-9 PMC8800281

[11] Tsuda M, Koga K, Chen T, Zhuo M. Neuronal and microglial mechanisms for neuropathic pain in the spinal dorsal horn and anterior cingulate cortex. J Neurochem. 2017;141(4):486–498. 28251660 10.1111/jnc.14001

[12] Chen T, Taniguchi W, Chen QY, Tozaki-Saitoh H, Song Q, Liu RH, Koga K, Matsuda T, Kaito-Sugimura Y, Wang J, et al. Top-down descending facilitation of spinal sensory excitatory transmission from the anterior cingulate cortex. Nat Commun. 2018;9(1):1886. 29760484 10.1038/s41467-018-04309-2 PMC5951839

[13] Kang SJ, Kwak C, Lee J, Sim S-E, Shim J, Choi T, Collingridge GL, Zhuo M, Kaang B-K. Bidirectional modulation of hyperalgesia via the specific control of excitatory and inhibitory neuronal activity in the ACC. Mol Brain. 2015;8(1):81. 26631249 10.1186/s13041-015-0170-6 PMC4668615

[14] Li XH, Matsuura T, Xue M, Chen QY, Liu RH, Lu JS, Shi W, Fan K, Zhou Z, Miao Z, et al. Oxytocin in the anterior cingulate cortex attenuates neuropathic pain and emotional anxiety by inhibiting presynaptic long-term potentiation. Cell Rep. 2021;36(3): Article 109411. 34289348 10.1016/j.celrep.2021.109411

[15] Auld F, Maschauer EL, Morrison I, Skene DJ, Riha RL. Evidence for the efficacy of melatonin in the treatment of primary adult sleep disorders. Sleep Med Rev. 2017;34:10–22. 28648359 10.1016/j.smrv.2016.06.005

[16] Buonfiglio D, Hummer DL, Armstrong A, Christopher Ehlen J, DeBruyne JP. Angelman syndrome and melatonin: What can they teach us about sleep regulation. J Pineal Res. 2020;69(4): Article e12697. 32976638 10.1111/jpi.12697 PMC7577950

[17] Ali T, Rehman SU, Shah FA, Kim MO. Acute dose of melatonin via Nrf2 dependently prevents acute ethanol-induced neurotoxicity in the developing rodent brain. J Neuroinflammation. 2018;15(1):119. 29679979 10.1186/s12974-018-1157-x PMC5911370

[18] Chaudhary S, Sahu U, Kar S, Parvez S. Phytanic acid-induced neurotoxicological manifestations and apoptosis ameliorated by mitochondria-mediated actions of melatonin. Mol Neurobiol. 2017;54(9):6960–6969. 27785753 10.1007/s12035-016-0209-4

[19] Pal S, Sahu A, Verma R, Haldar C. BPS-induced ovarian dysfunction: Protective actions of melatonin via modulation of SIRT-1/Nrf2/NFĸB and IR/PI3K/pAkt/GLUT-4 expressions in adult golden hamster. J Pineal Res. 2023;75(1): Article e12869. 37002642 10.1111/jpi.12869

[20] Chen C, Yang C, Wang J, Huang X, Yu H, Li S, Li S, Zhang Z, Liu J, Yang X, et al. Melatonin ameliorates cognitive deficits through improving mitophagy in a mouse model of Alzheimer’s disease. J Pineal Res. 2021;71(4): Article e12774. 34617321 10.1111/jpi.12774

[21] Melhuish Beaupre LM, Brown GM, Gonçalves VF, Kennedy JL. Melatonin’s neuroprotective role in mitochondria and its potential as a biomarker in aging, cognition and psychiatric disorders. Transl Psychiatry. 2021;11(1):339. 34078880 10.1038/s41398-021-01464-x PMC8172874

[22] Tamtaji OR, Reiter RJ, Alipoor R, Dadgostar E, Kouchaki E, Asemi Z. Melatonin and Parkinson disease: Current status and future perspectives for molecular mechanisms. Cell Mol Neurobiol. 2020;40(1):15–23. 31388798 10.1007/s10571-019-00720-5 PMC11448849

[23] Wang YS, Li YY, Cui W, Li LB, Zhang ZC, Tian BP, Zhang GS. Melatonin attenuates pain hypersensitivity and decreases astrocyte-mediated spinal neuroinflammation in a rat model of oxaliplatin-induced pain. Inflammation. 2017;40(6):2052–2061. 28812173 10.1007/s10753-017-0645-y

[24] Yu CX, Zhu B, Xu SF, Cao XD, Wu GC. The analgesic effects of peripheral and central administration of melatonin in rats. Eur J Pharmacol. 2000;403(1-2):49–53. 10969143 10.1016/s0014-2999(00)00421-0

[25] Ambriz-Tututi M, Granados-Soto V. Oral and spinal melatonin reduces tactile allodynia in rats via activation of MT2 and opioid receptors. Pain. 2007;132(3):273–280. 17346886 10.1016/j.pain.2007.01.025

[26] Lin JJ, Lin Y, Zhao TZ, Zhang CK, Zhang T, Chen XL, Ding JQ, Chang T, Zhang Z, Sun C, et al. Melatonin suppresses neuropathic pain via MT2-dependent and -independent pathways in dorsal root ganglia neurons of mice. Theranostics. 2017;7(7):2015–2032. 28656058 10.7150/thno.19500 PMC5485420

[27] Huang CT, Chen SH, Chang CF, Lin SC, Lue JH, Tsai YJ. Melatonin reduces neuropathic pain behavior and glial activation through MT_2_ melatonin receptor modulation in a rat model of lysophosphatidylcholine-induced demyelination neuropathy. Neurochem Int. 2020;140: Article 104827. 32853748 10.1016/j.neuint.2020.104827

[28] Zhang CK, Wang P, Ji YY, Zhao JS, Gu JX, Yan XX, Fan HW, Zhang MM, Qiao Y, Liu XD, et al. Potentiation of the lateral habenula-ventral tegmental area pathway underlines the susceptibility to depression in mice with chronic pain. Sci China Life Sci. 2024;67(1):67–82. 37864083 10.1007/s11427-023-2406-3

[29] Xu GY, Xu S, Zhang YX, Yu ZY, Zou F, Ma XS, Xia XL, Zhang WJ, Jiang JY, Song J. Cell-free extracts from human fat tissue with a hyaluronan-based hydrogel attenuate inflammation in a spinal cord injury model through M2 microglia/microphage polarization. Small. 2022;18(17): Article e2107838. 35333441 10.1002/smll.202107838

[30] Ao C, Jiao S, Wang Y, Yu L, Zou Q. Biological sequence classification: A review on data and general methods. Research. 2022;2022:0011. 39285948 10.34133/research.0011 PMC11404319

[31] Ahmad SB, Ali A, Bilal M, Rashid SM, Wani AB, Bhat RR, Rehman MU. Melatonin and health: Insights of melatonin action, biological functions, and associated disorders. Cell Mol Neurobiol. 2023;43(6):2437–2458. 36752886 10.1007/s10571-023-01324-w PMC9907215

[32] David S, Kroner A. Repertoire of microglial and macrophage responses after spinal cord injury. Nat Rev Neurosci. 2011;12(7):388–399. 21673720 10.1038/nrn3053

[33] Wang YY, Shen D, Zhao LJ, Zeng N, Hu TH. Sting is a critical regulator of spinal cord injury by regulating microglial inflammation via interacting with TBK1 in mice. Biochem Biophys Res Commun. 2019;517(4):741–748. 31400857 10.1016/j.bbrc.2019.07.125

[34] Tian Y, Bao Z, Ji Y, Mei X, Yang H. Epigallocatechin-3-gallate protects H_2_O_2_-induced nucleus pulposus cell apoptosis and inflammation by inhibiting cGAS/Sting/NLRP3 activation. Drug Des Devel Ther. 2020;14:2113–2122. 10.2147/DDDT.S251623 PMC7266312 32546974

[35] Sun J, Zhou YQ, Xu BY, Li JY, Zhang LQ, Li DY, Zhang S, Wu JY, Gao SJ, Ye DW, et al. STING/NF-κB/IL-6-mediated inflammation in microglia contributes to spared nerve injury (SNI)-induced pain initiation. J Neuroimmune Pharmacol. 2022;17(3-4):453–469. 34727296 10.1007/s11481-021-10031-6

[36] Arendt J, Deacon S. Treatment of circadian rhythm disorders—Melatonin. Chronobiol Int. 1997;14(2):185–204. 9095378 10.3109/07420529709001155

[37] Arendt J. Melatonin and human rhythms. Chronobiol Int. 2006;23(1-2):21–37. 16687277 10.1080/07420520500464361

[38] Skene DJ, Arendt J. Circadian rhythm sleep disorders in the blind and their treatment with melatonin. Sleep Med. 2007;8(6):651–655. 17420154 10.1016/j.sleep.2006.11.013

[39] Pandi-Perumal SR, BaHammam AS, Brown GM, Spence DW, Bharti VK, Kaur C, Hardeland R, Cardinali DP. Melatonin antioxidative defense: Therapeutical implications for aging and neurodegenerative processes. Neurotox Res. 2013;23(3):267–300. 22739839 10.1007/s12640-012-9337-4

[40] Zhang Z, Inserra PF, Liang B, Ardestani SK, Elliott KK, Molitor M, Watson RR. Melatonin, immune modulation and aging. Autoimmunity. 1997;26(1):43–53. 9556354 10.3109/08916939709009549

[41] Cui Y, Hong S, Xia Y, Li X, He X, Hu X, Li Y, Wang X, Lin K, Mao L. Melatonin engineering M2 macrophage-derived exosomes mediate endoplasmic reticulum stress and immune reprogramming for periodontitis therapy. Adv Sci. 2023;10(27): Article e2302029. 10.1002/advs.202302029 PMC10520618 37452425

[42] Bacon ER, Mishra A, Wang Y, Desai MK, Yin F, Brinton RD. Neuroendocrine aging precedes perimenopause and is regulated by DNA methylation. Neurobiol Aging. 2019;74:213–224. 30497015 10.1016/j.neurobiolaging.2018.09.029 PMC7117064

[43] Wilhelmsen M, Amirian I, Reiter RJ, Rosenberg J, Gögenur I. Analgesic effects of melatonin: A review of current evidence from experimental and clinical studies. J Pineal Res. 2011;51(3):270–277. 21615490 10.1111/j.1600-079X.2011.00895.x

[44] Andersen LPH, Gögenur I, Fenger AQ, Petersen MC, Rosenberg J, Werner MU. Analgesic and antihyperalgesic effects of melatonin in a human inflammatory pain model: A randomized, double-blind, placebo-controlled, three-arm crossover study. Pain. 2015;156(11):2286–2294. 26164585 10.1097/j.pain.0000000000000284

[45] Posa L, Lopez-Canul M, Rullo L, De Gregorio D, Dominguez-Lopez S, Kaba Aboud M, Caputi FF, Candeletti S, Romualdi P, Gobbi G. Nociceptive responses in melatonin MT2 receptor knockout mice compared to MT1 and double MT1 /MT2 receptor knockout mice. J Pineal Res. 2020;69(3): Article e12671. 32430930 10.1111/jpi.12671

[46] Bian XL, Qin C, Cai CY, Zhou Y, Tao Y, Lin YH, Wu HY, Chang L, Luo CX, Zhu DY. Anterior cingulate cortex to ventral hippocampus circuit mediates contextual fear generalization. J Neurosci. 2019;39(29):5728–5739. 31097621 10.1523/JNEUROSCI.2739-18.2019 PMC6636085

[47] Blom SM, Pfister JP, Santello M, Senn W, Nevian T. Nerve injury-induced neuropathic pain causes disinhibition of the anterior cingulate cortex. J Neurosci. 2014;34(17):5754–5764. 24760836 10.1523/JNEUROSCI.3667-13.2014 PMC6608297

[48] Zhao R, Zhou H, Huang L, Xie Z, Wang J, Gan WB, Yang G. Neuropathic pain causes pyramidal neuronal hyperactivity in the anterior cingulate cortex. Front Cell Neurosci. 2018;12:107. 29731710 10.3389/fncel.2018.00107 PMC5919951

[49] Meng XL, Fu P, Wang L, Yang X, Hong G, Zhao X, Lao J. Increased EZH2 levels in anterior cingulate cortex microglia aggravate neuropathic pain by inhibiting autophagy following brachial plexus avulsion in rats. Neurosci Bull. 2020;36(7):793–805. 32346844 10.1007/s12264-020-00502-w PMC7340721

[50] Long DD, Zhang YZ, Liu A, Shen L, Wei HR, Lou QQ, Hu SS, Chen DY, Chai XQ, Wang D. Microglia sustain anterior cingulate cortex neuronal hyperactivity in nicotine-induced pain. J Neuroinflammation. 2023;20(1):81. 36944965 10.1186/s12974-023-02767-0 PMC10031886

[51] Shao FB, Fang JF, Wang SS, Qiu MT, Xi DN, Jin XM, Liu JG, Shao XM, Shen Z, Liang Y, et al. Anxiolytic effect of GABAergic neurons in the anterior cingulate cortex in a rat model of chronic inflammatory pain. Mol Brain. 2021;14(1):139. 34507588 10.1186/s13041-021-00849-9 PMC8431944

[52] Lopez-Canul M, Palazzo E, Dominguez-Lopez S, Luongo L, Lacoste B, Comai S, Angeloni D, Fraschini F, Boccella S, Spadoni G, et al. Selective melatonin MT2 receptor ligands relieve neuropathic pain through modulation of brainstem descending antinociceptive pathways. Pain. 2015;156(2):305–317. 25599452 10.1097/01.j.pain.0000460311.71572.5f

[53] Posa L, De Gregorio D, Lopez-Canul M, He Q, Darcq E, Rullo L, Pearl-Dowler L, Luongo L, Candeletti S, Romualdi P, et al. Supraspinal melatonin MT2 receptor agonism alleviates pain via a neural circuit that recruits mu opioid receptors. J Pineal Res. 2022;73(4): Article e12825. 35996205 10.1111/jpi.12825

[54] Meeker TJ, Schmid AC, Keaser ML, Khan SA, Gullapalli RP, Dorsey SG, Greenspan JD, Seminowicz DA. Tonic pain alters functional connectivity of the descending pain modulatory network involving amygdala, periaqueductal gray, parabrachial nucleus and anterior cingulate cortex. NeuroImage. 2022;256: Article 119278. 35523367 10.1016/j.neuroimage.2022.119278 PMC9250649

[55] Lee JY, You T, Lee CH, Im GH, Seo H, Woo CW, Kim SG. Role of anterior cingulate cortex inputs to periaqueductal gray for pain avoidance. Curr Biol. 2022;32(13):2834–2847.e5. 35609604 10.1016/j.cub.2022.04.090

[56] Honjoh K, Nakajima H, Hirai T, Watanabe S, Matsumine A. Relationship of inflammatory cytokines from M1-type microglia/macrophages at the injured site and lumbar enlargement with neuropathic pain after spinal cord injury in the CCL21 knockout (plt) mouse. Front Cell Neurosci. 2019;13:525. 31824269 10.3389/fncel.2019.00525 PMC6881269

[57] Lv Z, Wang P, Li W, Xie Y, Sun W, Jin X, Jiang R, Fei Y, Liu Y, Shi T, et al. Bifunctional TRPV1 targeted magnetothermal switch to attenuate osteoarthritis progression. Research. 2024;7:0316. 38371274 10.34133/research.0316 PMC10871150

[58] Decout A, Katz JD, Venkatraman S, Ablasser A. The cGAS-STING pathway as a therapeutic target in inflammatory diseases. Nat Rev Immunol. 2021;21(9):548–569. 33833439 10.1038/s41577-021-00524-z PMC8029610

[59] Hu X, Zhang H, Zhang Q, Yao X, Ni W, Zhou K. Emerging role of STING signalling in CNS injury: Inflammation, autophagy, necroptosis, ferroptosis and pyroptosis. J Neuroinflammation. 2022;19(1):242. 36195926 10.1186/s12974-022-02602-y PMC9531511

[60] Wu W, Zhang X, Wang S, Li T, Hao Q, Li S, Yao W, Sun R. Pharmacological inhibition of the cGAS-STING signaling pathway suppresses microglial M1-polarization in the spinal cord and attenuates neuropathic pain. Neuropharmacology. 2022;217: Article 109206. 35926582 10.1016/j.neuropharm.2022.109206

[61] Chen C, Xu P. Cellular functions of cGAS-STING signaling. Trends Cell Biol. 2023;33(8):630–648. 36437149 10.1016/j.tcb.2022.11.001

[62] Ghislat G, Cheema AS, Baudoin E, Verthuy C, Ballester PJ, Crozat K, Attaf N, Dong C, Milpied P, Malissen B, et al. NF-κB-dependent IRF1 activation programs cDC1 dendritic cells to drive antitumor immunity. Sci Immunol. 2021;6(61): Article eabg3570. 34244313 10.1126/sciimmunol.abg3570

[63] Barrat FJ, Crow MK, Ivashkiv LB. Interferon target-gene expression and epigenomic signatures in health and disease. Nat Immunol. 2019;20(12):1574–1583. 31745335 10.1038/s41590-019-0466-2 PMC7024546

[64] Racz I, Nadal X, Alferink J, Baños JE, Rehnelt J, Martín M, Pintado B, Gutierrez-Adan A, Sanguino E, Bellora N, et al. Interferon-gamma is a critical modulator of CB_2_ cannabinoid receptor signaling during neuropathic pain. J Neurosci. 2008;28(46):12136–12145. 19005078 10.1523/JNEUROSCI.3402-08.2008 PMC3844840

[65] Tsuda M, Masuda T, Kitano J, Shimoyama H, Tozaki-Saitoh H, Inoue K. IFN-gamma receptor signaling mediates spinal microglia activation driving neuropathic pain. Proc Natl Acad Sci USA. 2009;106(19):8032–8037. 19380717 10.1073/pnas.0810420106 PMC2683100

[66] Tan PH, Ji J, Yeh CC, Ji RR. Interferons in pain and infections: Emerging roles in neuro-immune and neuro-glial interactions. Front Immunol. 2021;12: Article 783725. 34804074 10.3389/fimmu.2021.783725 PMC8602180

[67] Kann O, Almouhanna F, Chausse B. Interferon γ: A master cytokine in microglia-mediated neural network dysfunction and neurodegeneration. Trends Neurosci. 2022;45(12):913–927. 36283867 10.1016/j.tins.2022.10.007

[68] Sun J, Zhang Q, Liu X, Shang X. Downregulation of interferon-induced protein with tetratricopeptide repeats 3 relieves the inflammatory response and myocardial fibrosis of mice with myocardial infarction and improves their cardiac function. Exp Anim. 2021;70(4):522–531. 34234081 10.1538/expanim.21-0060 PMC8614010

[69] Huang C, Lewis C, Borg NA, Canals M, Diep H, Drummond GR, Goode RJ, Schittenhelm RB, Vinh A, Zhu M, et al. Proteomic identification of interferon-induced proteins with tetratricopeptide repeats as markers of M1 macrophage polarization. J Proteome Res. 2018;17(4):1485–1499. 29508616 10.1021/acs.jproteome.7b00828

[70] Gu JX, Wang J, Ma FJ, Liu MM, Chen SH, Wei Y, Xiao YF, Lv PY, Liu X, Qu JQ, et al. Rab11a in the spinal cord: An essential contributor to complete Freund’s adjuvant-induced inflammatory pain in mice. Mol Brain. 2023;16(1):70. 37770900 10.1186/s13041-023-01057-3 PMC10537208

[71] Saura J, Tusell JM, Serratosa J. High-yield isolation of murine microglia by mild trypsinization. Glia. 2003;44(3):183–189. 14603460 10.1002/glia.10274

[72] Cheng J, Zhang R, Xu Z, Ke Y, Sun R, Yang H, Zhang X, Zhen X, Zheng LT. Early glycolytic reprogramming controls microglial inflammatory activation. J Neuroinflammation. 2021;18(1):129. 34107997 10.1186/s12974-021-02187-y PMC8191212

[73] Liu M, Li X, Wang J, Ji Y, Gu J, Wei Y, Peng L, Tian C, Lv P, Wang P, et al. Identification and validation of Rab11a in rat orofacial inflammatory pain model induced by CFA. Neurochem Int. 2023;168: Article 105550. 37268020 10.1016/j.neuint.2023.105550

[74] Deng B, Liao F, Liu Y, He P, Wei S, Liu C, Dong W. Comprehensive analysis of endoplasmic reticulum stress-associated genes signature of ulcerative colitis. Front Immunol. 2023;14:1158648. 37287987 10.3389/fimmu.2023.1158648 PMC10243217

