# Supplementary material for: Melatonin Induces Analgesic Effects through MT2 Receptor-Mediated Neuroimmune Modulation in the Mice Anterior Cingulate Cortex
Source: Research (Wash D C). 2024 Oct 8;7:0493. doi: 10.34133/research.0493 (PMC11458856; doi:10.34133/research.0493)
Supplement: Supplementary 1 — Figs. S1 to S3 Table S1 [file research.0493.f1.zip › Supplementary Table.docx]

Supplementary Table 1. Statistical analyses related to Figures. 1, 4-7 and Supplementary Figure 2.

| **Figure** | **Conditions and Sample size** | **Analysis** | **t or F value** | ***P* value** |
| --- | --- | --- | --- | --- |
| Fig. 1 B | Sham+saline, n = 6; SNI+saline, n = 6 | Two-Way ANOVA with Bonferroni’s post hoc analysis | F (1, 10) = 160.6 | *P* < 0.0001 |
|  | SNI+saline, n = 6; SNI+MLT (0.1 mg/kg), n = 6 | Two-Way ANOVA with Bonferroni’s post hoc analysis | F (1, 10) = 0.138 | *P* = 0.7181 |
|  | SNI+saline, n = 6; SNI+MLT (0.3 mg/kg), n = 6 | Two-Way ANOVA with Bonferroni’s post hoc analysis | F (1, 10) = 4.432 | *P* = 0.0615 |
|  | SNI+saline, n = 6; SNI+MLT (1 mg/kg), n = 6 | Two-Way ANOVA with Bonferroni’s post hoc analysis | F (1, 10) = 37.59 | *P* = 0.0001 |
|  | SNI+saline, n = 6; SNI+MLT (3 mg/kg), n = 6 | Two-Way ANOVA with Bonferroni’s post hoc analysis | F (1, 10) = 70.52 | *P* < 0.0001 |
|  | SNI+saline, n = 6; SNI+MLT (10 mg/kg), n = 6 | Two-Way ANOVA with Bonferroni’s post hoc analysis | F (1, 10) = 278.2 | *P* < 0.0001 |
| Fig. 1 D | Sham+saline, n = 6; SNI+saline, n = 6 | Two-Way ANOVA with Bonferroni’s post hoc analysis | F (1, 10) = 221.3 | *P* < 0.0001 |
|  | SNI+saline, n = 6; SNI+MLT (1.114 mg/kg), n = 6 | Two-Way ANOVA with Bonferroni’s post hoc analysis | F (1, 10) = 90.07 | *P* < 0.0001 |
|  | SNI+saline, n = 6; SNI+8-MP (7.5 mg/kg), n = 6 | Two-Way ANOVA with Bonferroni’s post hoc analysis | F (1, 10) = 51.02 | *P* < 0.0001 |
|  | SNI+saline, n = 6; SNI+Ramelteon (10 mg/kg) +4PP (2.5 mg/kg), n = 6 | Two-Way ANOVA with Bonferroni’s post hoc analysis | F (1, 10) = 1.258 | *P* = 0.2882 |
|  | SNI+MLT (1.114 mg/kg); SNI+8-MP (7.5 mg/kg), n = 6 | Two-Way ANOVA with Bonferroni’s post hoc analysis | F (1, 10) = 1.212 | *P* = 0.2966 |
| Fig. 1 E | Sham+saline, n = 10; SNI+saline, n = 10 | Two-Way ANOVA with Bonferroni’s post hoc analysis | F (1, 18) = 162.1 | *P* < 0.0001 |
|  | SNI+saline, n = 10; SNI+MLT (1 µM), n = 10 | Two-Way ANOVA with Bonferroni’s post hoc analysis | F (1, 18) = 0.0536 | *P* = 0.8195 |
|  | SNI+saline, n = 10; SNI+MLT (3 µM), n = 10 | Two-Way ANOVA with Bonferroni’s post hoc analysis | F (1, 18) = 0.6933 | *P* = 0.4160 |
|  | SNI+saline, n = 10; SNI+MLT (10 µM), n = 10 | Two-Way ANOVA with Bonferroni’s post hoc analysis | F (1, 18) = 8.430 | *P* = 0.0095 |
|  | SNI+saline, n = 10; SNI+MLT (30 µM), n = 10 | Two-Way ANOVA with Bonferroni’s post hoc analysis | F (1, 18) = 26.63 | *P* < 0.0001 |
|  | SNI+saline, n = 10; SNI+MLT (100 µM), n = 10 | Two-Way ANOVA with Bonferroni’s post hoc analysis | F (1, 18) = 61.32 | *P* < 0.0001 |
| Fig. 1 G | Sham+saline, n = 10; SNI+saline, n = 10 | Two-Way ANOVA with Bonferroni’s post hoc analysis | F (1, 18) = 168.8 | *P* < 0.0001 |
|  | SNI+saline, n = 10; SNI+MLT (13.33 µM), n = 10 | Two-Way ANOVA with Bonferroni’s post hoc analysis | F (1, 18) = 34.32 | *P* < 0.0001 |
|  | SNI+saline, n = 10; SNI+8-MP (10 µM), n = 10 | Two-Way ANOVA with Bonferroni’s post hoc analysis | F (1, 18) = 14.96 | *P* < 0.0001 |
|  | SNI+saline, n = 10; SNI+Ramelteon (10 µM) +4PP (10 µM), n = 10 | Two-Way ANOVA with Bonferroni’s post hoc analysis | F (1, 18) = 0.2704 | *P* = 0.6094 |
|  | SNI+MLT (13.33 µM), n = 10; SNI+8-MP (10 µM), n = 10 | Two-Way ANOVA with Bonferroni’s post hoc analysis | F (1, 18) = 1.179 | *P* = 0.2919 |
| Fig. 1 H | Sham+saline, n = 3; Sham+MLT(1.114 mg/kg), n = 3; SNI+saline, n = 3; SNI+MLT(1.114 mg/kg), n = 3 | One-Way ANOVA with Dunnett’s post hoc analysis | F (3, 8) = 1 | *P* = 0.3159 |
| Fig. 1 I | Sham+saline, n = 3; Sham+MLT(1.114 mg/kg), n = 3; SNI+saline, n = 3; SNI+MLT(1.114 mg/kg), n = 3 | One-Way ANOVA with Dunnett’s post hoc analysis | F (3, 8) = 8.076 | *P* = 0.0084 |
|  | SNI+saline *vs.* SNI+MLT |  |  | *P* = 0.0331 |
| Fig. 4E | Frequency: Sham, n = 6 cells in 3 mice; SNI, n = 6 cells in 3 mice | Unpaired t test | t = 2.611 | *P* = 0.0260 |
|  | Amplitude: Sham, 6 cells in 3 mice; SNI, n = 6 cells in 3 mice | Unpaired t test | t = 2.349 | *P* = 0.0407 |
| Fig. 4F | Baseline, n = 6 cells in 3 mice; MLT (13.33 μM), n = 6 cells in 3 mice; ACSF cleaning, n = 6 cells in 3 mice | One-Way ANOVA with Dunnett’s post hoc analysis | F (2, 15) = 5.877 | *P* = 0.0130 |
|  | MLT treatment *vs*. Baseline |  |  | *P* = 0.0143 |
| Fig. 4G | Baseline, n = 6 cells in 3 mice; MLT (13.33 μM), n = 6 cells in 3 mice; ACSF cleaning, n = 6 cells in 3 mice | One-Way ANOVA with Dunnett’s post hoc analysis | F (2, 15) = 0.7689 | *P* = 0.4810 |
|  | MLT treatment *vs*. Baseline |  |  | *P* = 0.6605 |
| Fig. 4I | Frequency: Sham, n = 7 cells in 4 mice; SNI, n = 6 cells in 3 mice | Unpaired t test | t = 1.807 | *P* = 0.098 |
|  | Amplitude: Sham, n = 7 cells in 4 mice; SNI, n = 6 cells in 3 mice | Unpaired t test | t = 1.638 | *P* = 0.130 |
| Fig. 4J | Baseline, n = 7 cells in 4 mice; MLT (13.33 μM), n = 7 cells in 4 mice; ACSF cleaning, n = 7 cells in 4 mice | One-Way ANOVA with Dunnett’s post hoc analysis | F (2, 18) = 0.1480 | *P* = 0.8635 |
| Fig. 4K | Baseline, n = 7 cells in 4 mice; MLT (13.33 μM), n = 7 cells in 4 mice; ACSF cleaning, n = 7 cells in 4 mice | One-Way ANOVA with Dunnett’s post hoc analysis | F (2, 18) = 0.8255 | *P* = 0.4539 |
| Fig. 4M | Sham+ACSF, n = 9 cells in 5 mice; Sham+MLT(13.33 μM), n = 9 cells in 5 mice; SNI+ACSF, n = 8 cells in 4 mice; SNI+MLT(13.33 μM), n = 10 cells in 5 mice | One-Way ANOVA with Dunnett’s post hoc analysis | F (3, 32) = 5.486 | *P* = 0.0037 |
|  | SNI+ACSF *vs*. Sham+ACSF |  |  | *P* = 0.0403 |
|  | SNI+MLT *vs*. SNI+ACSF |  |  | *P* = 0.0024 |
| Fig. 5 A | Sham+ACSF, n = 6 cells in 3 mice ; SNI+ACSF, n = 8 cells in 4 mice; | Two-Way ANOVA with Bonferroni’s post hoc analysis | F (1, 12) = 10. 79 | *P* = 0.0065 |
|  | SNI+ACSF, n = 8 cells in 4 mice; SNI+MLT(13.33 μM), n = 7 cells in 4 mice | Two-Way ANOVA with Bonferroni’s post hoc analysis | F (1, 13) = 6.880 | *P* = 0.0211 |
| Fig. 5 B | Sham+ACSF, n = 5 cells in 3 mice; SNI+ACSF, n = 7 cells in 4 mice; | Two-Way ANOVA with Bonferroni’s post hoc analysis | F (1, 10) = 0.046 | *P* = 0.8340 |
|  | SNI+ACSF, n = 7 cells in 4 mice; SNI+MLT(13.33 μM), n = 7 cells in 4 mice | Two-Way ANOVA with Bonferroni’s post hoc analysis | F (1, 11) = 0.0085 | *P* = 0.9279 |
| Fig. 5 D | Sham+ACSF, n = 6 cells in 3 mice; Sham+MLT(13.33 μM), n = 5 cells in 3 mice; SNI+ACSF, n = 8 cells in 4 mice; SNI+MLT(13.33 μM), n = 7 cells in 4 mice | One-Way ANOVA with Dunnett’s post hoc analysis | F (3, 22) = 4.093 | *P* = 0.0189 |
|  | Sham+ACSF *vs*. SNI+ACSF |  |  | *P* = 0.0180 |
|  | SNI+ACSF *vs*. SNI+MLT |  |  | *P* = 0.0404 |
| Fig. 5 E | Sham+ACSF, n = 6 cells in 3 mice; Sham+MLT(13.33 μM), n = 5 cells in 3 mice; SNI+ACSF, n = 8 cells in 4 mice; SNI+MLT(13.33 μM), n = 7 cells in 4 mice | One-Way ANOVA with Dunnett’s post hoc analysis | F (3, 22) = 8.240 | *P* = 0.0007 |
|  | Sham+ACSF *vs*. SNI+ACSF |  |  | *P* = 0.0005 |
|  | SNI+ACSF *vs*. SNI+MLT |  |  | *P* = 0.0035 |
| Fig. 5 G | Sham+ACSF, n = 5 cells in 3 mice; Sham+MLT(13.33 μM), n = 5 cells in 3 mice; SNI+ACSF, n = 7 cells in 4 mice; SNI+MLT(13.33 μM), n = 7 cells in 4 mice | One-Way ANOVA with Dunnett’s post hoc analysis | F (3, 20) = 4.093 | *P* = 0.0636 |
|  | Sham+ACSF *vs*. SNI+ACSF |  |  | *P* = 0.0256 |
|  | SNI+ACSF *vs*. SNI+MLT |  |  | *P* = 0.6991 |
| Fig. 5 H | Sham+ACSF, n = 5 cells in 3 mice; Sham+MLT(13.33 μM), n = 5 cells in 3 mice; SNI+ACSF, n = 7 cells in 4 mice; SNI+MLT(13.33 μM), n = 7 cells in 4 mice | One-Way ANOVA with Dunnett’s post hoc analysis | F (3, 20) = 5.759 | *P* = 0.0052 |
|  | Sham+ACSF *vs*. SNI+ACSF |  |  | *P* = 0.0039 |
|  | SNI+ACSF *vs*. SNI+MLT |  |  | *P* = 0.1562 |
| Fig. 5 I | Sham+saline, n = 3; Sham+MLT(1.114 mg/kg), n = 3; SNI+saline, n = 3; SNI+MLT(1.114 mg/kg), n = 3 | One-Way ANOVA with Dunnett’s post hoc analysis | F (3, 8) = 9.867 | *P* = 0.0046 |
|  | Sham+saline *vs*. SNI+saline |  |  | *P* = 0.0106 |
|  | SNI+saline *vs*. SNI+MLT |  |  | *P* = 0.0109 |
| Fig. 5 J | Sham+saline, n = 3; Sham+MLT(1.114 mg/kg), n = 3; SNI+saline, n = 3; SNI+MLT(1.114 mg/kg), n = 3 | One-Way ANOVA with Dunnett’s post hoc analysis | F (3, 8) = 20.13 | *P* = 0.0004 |
|  | Sham+saline *vs*. SNI+saline |  |  | *P* = 0.0004 |
|  | SNI+saline *vs*. SNI+MLT |  |  | *P* = 0.0025 |
| Fig. 6 D | Control, n = 3; LPS 3 h, n = 3; LPS 6 h, n = 3; LPS 12 h, n = 3; LPS 24 h, n = 3 | One-Way ANOVA with Dunnett’s post hoc analysis | F (4, 10) = 116.5 | *P* < 0.0001 |
|  | Control *vs.* LPS 3 h |  |  | *P* < 0.0001 |
|  | Control *vs.* LPS 6 h |  |  | *P* < 0.0001 |
|  | Control *vs.* LPS 12 h |  |  | *P* < 0.0001 |
|  | Control *vs.* LPS 24 h |  |  | *P* < 0.0001 |
| Fig. 6 E | Control, n = 3; LPS 3 h, n = 3; LPS 6 h, n = 3; LPS 12 h, n = 3; LPS 24 h, n = 3 | One-Way ANOVA with Dunnett’s post hoc analysis | F (4, 10) = 9.273 | *P* = 0.0021 |
|  | Control *vs.* LPS 3 h |  |  | *P* = 0.0565 |
|  | Control *vs.* LPS 6 h |  |  | *P* = 0.0120 |
|  | Control *vs.* LPS 12 h |  |  | *P* = 0.0033 |
|  | Control *vs.* LPS 24 h |  |  | *P* = 0.0007 |
| Fig. 6 F | Control, n = 3; LPS 3 h, n = 3; LPS 6 h, n = 3; LPS 12 h, n = 3; LPS 24 h, n = 3 | One-Way ANOVA with Dunnett’s post hoc analysis | F (4, 10) = 3801 | *P* < 0.0001 |
|  | Control *vs.* LPS 3 h |  |  | *P* = 0.0020 |
|  | Control *vs.* LPS 6 h |  |  | *P* < 0.0001 |
|  | Control *vs.* LPS 12 h |  |  | *P* < 0.0001 |
|  | Control *vs.* LPS 24 h |  |  | *P* < 0.0001 |
| Fig. 6 G | Control, n = 3; LPS 3 h, n = 3; LPS 6 h, n = 3; LPS 12 h, n = 3; LPS 24 h, n = 3 | One-Way ANOVA with Dunnett’s post hoc analysis | F (4, 10) = 16.40 | *P* = 0.0002 |
|  | Control *vs.* LPS 3 h |  |  | *P* = 0.9879 |
|  | Control *vs.* LPS 6 h |  |  | *P* = 0.0003 |
|  | Control *vs.* LPS 12 h |  |  | *P* = 0.0045 |
|  | Control *vs.* LPS 24 h |  |  | *P* = 0.0018 |
| Fig. 6 H | Control, n = 3; LPS 12 h, n = 3; LPS 12 h+MLT, n = 3 | One-Way ANOVA with Dunnett’s post hoc analysis | F (2, 6) = 44.45 | *P* = 0.0003 |
|  | Control *vs*. LPS 12 h |  |  | *P* = 0.0002 |
|  | LPS 12 h *vs*. LPS 12 h+MLT |  |  | *P* = 0.0354 |
| Fig. 6 I | Control, n = 3; LPS 12 h, n = 3; LPS 12 h+MLT, n = 3 | One-Way ANOVA with Dunnett’s post hoc analysis | F (2, 6) = 21.70 | *P* = 0.0018 |
|  | Control *vs*. LPS 12 h |  |  | *P* = 0.0023 |
|  | LPS 12 h *vs*. LPS 12 h+MLT |  |  | *P* = 0.9991 |
| Fig. 6 J | Control, n = 3; LPS 12 h, n = 3; LPS 12 h+MLT, n = 3 | One-Way ANOVA with Dunnett’s post hoc analysis | F (2, 6) = 19.16 | *P* = 0.0025 |
|  | Control *vs*. LPS 12 h |  |  | *P* = 0.0035 |
|  | LPS 12 h *vs*. LPS 12 h+MLT |  |  | *P* = 0.0028 |
| Fig. 6 K | Control, n = 3; LPS 12 h, n = 3; LPS 12 h+MLT, n = 3 | One-Way ANOVA with Dunnett’s post hoc analysis | F (2, 6) = 54.66 | *P* = 0.0001 |
|  | Control *vs*. LPS 12 h |  |  | *P* = 0.0002 |
|  | LPS 12 h *vs*. LPS 12 h+MLT |  |  | *P* = 0.0002 |
| Fig. 6 M |  |  |  |  |
| CD16/32 | Control, n = 3; LPS 12 h, n = 3; LPS 12 h+MLT, n = 3 | One-Way ANOVA with Dunnett’s post hoc analysis | F (2, 6) = 13.57 | *P* = 0.0059 |
|  | Control *vs*. LPS 12 h |  |  | *P* = 0.0238 |
|  | LPS 12 h *vs*. LPS 12 h+MLT |  |  | *P* = 0.0040 |
| iNOS | Control, n = 3; LPS 12 h, n = 3; LPS 12 h+MLT, n = 3 | One-Way ANOVA with Dunnett’s post hoc analysis | F (2, 6) = 25.86 | *P* = 0.0011 |
|  | Control *vs*. LPS 12 h |  |  | *P* = 0.0248 |
|  | LPS 12 h *vs*. LPS 12 h+MLT |  |  | *P* = 0.0007 |
| ARG-1 | Control, n = 3; LPS 12 h, n = 3; LPS 12 h+MLT, n = 3 | One-Way ANOVA with Dunnett’s post hoc analysis | F (2, 6) = 12.29 | *P* = 0.0076 |
|  | Control *vs*. LPS 12 h |  |  | *P* = 0.0126 |
|  | LPS 12 h *vs*. LPS 12 h+MLT |  |  | *P* = 0.0071 |
| CD206 | Control, n = 3; LPS 12 h, n = 3; LPS 12 h+MLT, n = 3 | One-Way ANOVA with Dunnett’s post hoc analysis | F (2, 6) = 12.39 | *P* = 0.0074 |
|  | Control *vs*. LPS 12 h |  |  | *P* = 0.1550 |
|  | LPS 12 h *vs*. LPS 12 h+MLT |  |  | *P* = 0.0450 |
| Fig. 7A | Sham+saline, n = 3; Sham+MLT(1.114 mg/kg), n = 3; SNI+saline, n = 3; SNI+MLT(1.114 mg/kg), n = 3 | One-Way ANOVA with Dunnett’s post hoc analysis | F (3, 8) = 11.35 | *P* = 0.0030 |
|  | Sham+saline *vs*. SNI+saline |  |  | *P* = 0.0109 |
|  | SNI+saline *vs*. SNI+MLT |  |  | *P* = 0.0088 |
| Fig. 7B | Sham+saline, n = 3; Sham+MLT(1.114 mg/kg), n = 3; SNI+saline, n = 3; SNI+MLT(1.114 mg/kg), n = 3 | One-Way ANOVA with Dunnett’s post hoc analysis | F (3, 8) = 5.756 | *P* = 0.0214 |
|  | Sham+saline *vs*. SNI+saline |  |  | *P* = 0.0232 |
|  | SNI+saline *vs*. SNI+MLT |  |  | *P* = 0.0378 |
| Fig. 7C | Sham+saline, n = 3; Sham+MLT(1.114 mg/kg), n = 3; SNI+saline, n = 3; SNI+MLT(1.114 mg/kg), n = 3 | One-Way ANOVA with Dunnett’s post hoc analysis | F (3, 8) = 11.56 | *P* = 0.0028 |
|  | Sham+saline *vs*. SNI+saline |  |  | *P* = 0.0068 |
|  | SNI+saline *vs*. SNI+MLT |  |  | *P* = 0.0013 |
| Fig. 7D | Sham+saline, n = 3; Sham+MLT(1.114 mg/kg), n = 3; SNI+saline, n = 3; SNI+MLT(1.114 mg/kg), n = 3 | One-Way ANOVA with Dunnett’s post hoc analysis | F (3, 8) = 4.740 | *P* = 0.0349 |
|  | Sham+saline *vs*. SNI+saline |  |  | *P* = 0.9903 |
|  | SNI+saline *vs*. SNI+MLT |  |  | *P* = 0.0242 |
| Fig. 7E | Sham+saline, n = 4; Sham+MLT(1.114 mg/kg), n = 4; SNI+saline, n = 4; SNI+MLT(1.114 mg/kg), n = 4 | One-Way ANOVA with Dunnett’s post hoc analysis | F (3, 12) = 8.975 | *P* = 0.0022 |
|  | Sham+saline *vs*. SNI+saline |  |  | *P* = 0.0016 |
|  | SNI+saline *vs*. SNI+MLT |  |  | *P* = 0.0041 |
| Fig. 7F | Sham+saline, n = 4; Sham+MLT(1.114 mg/kg), n = 4; SNI+saline, n = 4; SNI+MLT(1.114 mg/kg), n = 4 | One-Way ANOVA with Dunnett’s post hoc analysis | F (3, 12) = 10.20 | *P* = 0.0013 |
|  | Sham+saline *vs*. SNI+saline |  |  | *P* = 0.0005 |
|  | SNI+saline *vs*. SNI+MLT |  |  | *P* = 0.0079 |
| Fig. 7G | Sham+saline, n = 4; Sham+MLT(1.114 mg/kg), n = 4; SNI+saline, n = 4; SNI+MLT(1.114 mg/kg), n = 4 | One-Way ANOVA with Dunnett’s post hoc analysis | F (3, 12) = 6.089 | *P* = 0.0184 |
|  | Sham+saline *vs*. SNI+saline |  |  | *P* = 0.0248 |
|  | SNI+saline *vs*. SNI+MLT |  |  | *P* = 0.0139 |
| Fig. 7H | Sham+saline, n = 4; Sham+MLT(1.114 mg/kg), n = 4; SNI+saline, n = 4; SNI+MLT(1.114 mg/kg), n = 4 | One-Way ANOVA with Dunnett’s post hoc analysis | F (3, 12) = 9.978 | *P* = 0.0014 |
|  | Sham+saline *vs*. SNI+saline |  |  | *P* = 0.0275 |
|  | SNI+saline *vs*. SNI+MLT |  |  | *P* = 0.0006 |
| Fig. 7K | Sham+saline, n = 3; Sham+MLT(1.114 mg/kg), n = 3; SNI+saline, n = 3; SNI+MLT(1.114 mg/kg), n = 3 | One-Way ANOVA with Dunnett’s post hoc analysis | F (3, 8) = 33.49 | *P* < 0.0001 |
|  | Sham+saline *vs*. SNI+saline |  |  | *P* < 0.0001 |
|  | SNI+saline *vs*. SNI+MLT |  |  | *P* = 0.0002 |
| Fig. 7L | Sham+saline, n = 3; Sham+MLT(1.114 mg/kg), n = 3; SNI+saline, n = 3; SNI+MLT(1.114 mg/kg), n = 3 | One-Way ANOVA with Dunnett’s post hoc analysis | F (3, 8) = 13.69 | *P* = 0.0016 |
|  | Sham+saline *vs*. SNI+saline |  |  | *P* = 0.0012 |
|  | SNI+saline *vs*. SNI+MLT |  |  | *P* = 0.0019 |
| Fig. 7M | Sham+saline, n = 3; Sham+MLT(1.114 mg/kg), n = 3; SNI+saline, n = 3; SNI+MLT(1.114 mg/kg), n = 3 | One-Way ANOVA with Dunnett’s post hoc analysis | F (3, 8) = 5.864 | *P* = 0.0203 |
|  | Sham+saline *vs*. SNI+saline |  |  | *P* = 0.0271 |
|  | SNI+saline *vs*. SNI+MLT |  |  | *P* = 0.0351 |
| Fig. 7N | Sham+saline, n = 3; Sham+MLT(1.114 mg/kg), n = 3; SNI+saline, n = 3; SNI+MLT(1.114 mg/kg), n = 3 | One-Way ANOVA with Dunnett’s post hoc analysis | F (3, 8) = 11.19 | *P* = 0.0031 |
|  | Sham+saline *vs*. SNI+saline |  |  | *P* = 0.0070 |
|  | SNI+saline *vs*. SNI+MLT |  |  | *P* = 0.0021 |
| Fig. S1B | Sham+saline, n=8; SNI+saline, n=8; SNI+MLT, n=8 | Two-Way ANOVA with Bonferroni’s post hoc analysis | F (2, 21) = 18.05 | *P* < 0.0001 |
|  | Sham+saline *vs*. SNI+saline |  |  | *P* < 0.0001 |
|  | SNI+saline *vs*. SNI+MLT |  |  | *P* < 0.001 |
| Fig. S1C | Sham+saline (Pre, n=6) *vs*. SNI+saline (Pre, n=6) | Student t test | t = 4.070 | *P = 0.0023* |
|  | SNI+MLT (Post, n=6) *vs*. SNI+MLT (Pre, n=6) | Student t test | t = 2.307 | *P = 0.0437* |
| Fig. S3B | Sham+ACSF, n = 6 cells in 3 mice; Sham+MLT(13.33 μM), n = 5 cells in 3 mice; SNI+ACSF, n = 8 cells in 4 mice; SNI+MLT(13.33 μM), n = 7 cells in 4 mice | One-Way ANOVA with Tukey’s post hoc analysis | F (3, 22) = 0.884 | *P* = 0.4647 |
| Fig. S3C | Sham+ACSF, n = 6 cells in 3 mice; Sham+MLT(13.33 μM), n = 5 cells in 3 mice; SNI+ACSF, n = 8 cells in 4 mice; SNI+MLT(13.33 μM), n = 7 cells in 4 mice | One-Way ANOVA with Tukey’s post hoc analysis | F (3, 22) = 2.473 | *P* = 0.0884 |
| Fig. S3D | Sham+ACSF, n = 6 cells in 3 mice; Sham+MLT(13.33 μM), n = 5 cells in 3 mice; SNI+ACSF, n = 8 cells in 4 mice; SNI+MLT(13.33 μM), n = 7 cells in 4 mice | One-Way ANOVA with Tukey’s post hoc analysis | F (3, 22) = 4.426 | *P* = 0.0140 |
|  | SNI+MLT *vs*. SNI+MLT |  |  | *P =* 0.0074 |
| Fig. S3E | Sham+ACSF, n = 6 cells in 3 mice; Sham+MLT(13.33 μM), n = 5 cells in 3 mice; SNI+ACSF, n = 8 cells in 4 mice; SNI+MLT(13.33 μM), n = 7 cells in 4 mice | One-Way ANOVA with Tukey’s post hoc analysis | F (3, 22) = 6.997 | *P* = 0.0018 |
|  | Sham+ACSF *vs*. SNI+MLT |  |  | *P =* 0.0443 |
|  | Sham+MLT *vs*. SNI+MLT |  |  | *P =* 0.0011 |
| Fig. S3F | Sham+ACSF, n = 6 cells in 3 mice; Sham+MLT(13.33 μM), n = 5 cells in 3 mice; SNI+ACSF, n = 8 cells in 4 mice; SNI+MLT(13.33 μM), n = 7 cells in 4 mice | One-Way ANOVA with Tukey’s post hoc analysis | F (3, 22) = 8.520 | *P* = 0.0006 |
|  | Sham+ACSF *vs*. SNI+ACSF |  |  | *P* = 0.0074 |
|  | Sham+ACSF *vs*. SNI+MLT |  |  | *P* = 0.0024 |
|  | Sham+MLT *vs*. SNI+ACSF |  |  | *P* = 0.0358 |
|  | Sham+MLT *vs*. SNI+MLT |  |  | *P* = 0.0125 |
| Fig. S3G | Sham+ACSF, n = 6 cells in 3 mice; Sham+MLT(13.33 μM), n = 5 cells in 3 mice; SNI+ACSF, n = 8 cells in 4 mice; SNI+MLT(13.33 μM), n = 7 cells in 4 mice | One-Way ANOVA with Tukey’s post hoc analysis | F (3, 22) = 2.508 | *P* = 0.0854 |
| Fig. S3H | Sham+ACSF, n = 6 cells in 3 mice; Sham+MLT(13.33 μM), n = 5 cells in 3 mice; SNI+ACSF, n = 8 cells in 4 mice; SNI+MLT(13.33 μM), n = 7 cells in 4 mice | One-Way ANOVA with Tukey’s post hoc analysis | F (3, 22) = 6.644 | *P* = 0.0023 |
|  | Sham+MLT *vs*. SNI+ACSF |  |  | *P* = 0.0095 |
|  | Sham+MLT *vs*. SNI+MLT |  |  | *P* = 0.0084 |
